# Neuroinflammation in Alzheimer’s Disease

**DOI:** 10.3390/biomedicines9050524

**Published:** 2021-05-07

**Authors:** Isaac G. Onyango, Gretsen V. Jauregui, Mária Čarná, James P. Bennett, Gorazd B. Stokin

**Affiliations:** 1 Centre for Translational Medicine International Clinical Research Centre, St. Anne’s University Hospital, CZ-65691 Brno, Czech Republic; gretsen.velezmoro@fnusa.cz (G.V.J.); maria.carna@fnusa.cz (M.Č.); 2 Neurodegeneration Therapeutics, 3050A Berkmar Drive, Charlottesville, VA 22901, USA; jpb8u@icloud.com; 3 Translational Aging and Neuroscience Program, Mayo Clinic, 200 First St. SW, Rochester, MN 55905, USA; 4 Division of Neurology, University Medical Centre, Zaloška cesta 2, 1000 Ljubljana, Slovenia

**Keywords:** Alzheimer’s disease, neuroinflammation, immunosenescence, inflammasome, mitochondria, microglia, astrocytes, DAMPs, SASP

## Abstract

Alzheimer’s disease (AD) is a neurodegenerative disease associated with human aging. Ten percent of individuals over 65 years have AD and its prevalence continues to rise with increasing age. There are currently no effective disease modifying treatments for AD, resulting in increasingly large socioeconomic and personal costs. Increasing age is associated with an increase in low-grade chronic inflammation (inflammaging) that may contribute to the neurodegenerative process in AD. Although the exact mechanisms remain unclear, aberrant elevation of reactive oxygen and nitrogen species (RONS) levels from several endogenous and exogenous processes in the brain may not only affect cell signaling, but also trigger cellular senescence, inflammation, and pyroptosis. Moreover, a compromised immune privilege of the brain that allows the infiltration of peripheral immune cells and infectious agents may play a role. Additionally, meta-inflammation as well as gut microbiota dysbiosis may drive the neuroinflammatory process. Considering that inflammatory/immune pathways are dysregulated in parallel with cognitive dysfunction in AD, elucidating the relationship between the central nervous system and the immune system may facilitate the development of a safe and effective therapy for AD. We discuss some current ideas on processes in inflammaging that appear to drive the neurodegenerative process in AD and summarize details on a few immunomodulatory strategies being developed to selectively target the detrimental aspects of neuroinflammation without affecting defense mechanisms against pathogens and tissue damage.

## 1. Introduction

Aging is characterized by dysregulated immune [1] and metabolic homeostasis [2,3] where there is chronic sterile low-grade inflammation or inflammaging [4] that involves cellular senescence [5,6], immunosenescence [7,8,9,10], mitochondrial dysfunction [11,12], defective autophagy [13,14] and mitophagy [15,16], dysregulation of the ubiquitin–proteasome system [17,18], activation of the DNA damage response [19,20], meta-inflammation or metaflammation from chronic overnutrition or obesity [21,22], and gut microbiota dysbiosis [5,23,24,25]. These are reflected by changes in circulating immune markers including C-reactive protein (CRP) [26], interleukin-6 (IL-6) [27], tumor necrosis factor alpha (TNF-α) [28] and its soluble receptors (tumor necrosis factor receptor I (TNFR-I) and tumor necrosis factor receptor II (TNFR-II)) [28], vascular cell adhesion molecule I (VCAM-I) [29], d-dimer [30], and sirtuin signaling [31,32]. The drawback of chronic subclinical inflammation is that it is an essential risk factor for increasing the incidence of degenerative diseases such as AD [33,34,35]. There are currently an estimated 728 million persons aged 65 years or over in the world. In the next 30 years, this number is expected to more than double to exceed 1.5 billion in 2050 (https://www.un.org/development/desa/pd/news/world-population-ageing-2020-highlights (accessed on 20 March 2021)). Thus, the aging population vulnerable to inflammaging will significantly increase over the next few decades.

AD is a chronic devastating neurodegenerative disorder in which increasing age is the strongest non-modifiable disease risk factor [36,37]. There are currently no effective therapies for AD [38,39]. It is clinically characterized by the progressive deterioration of memory and other cognitive functions [40]. It is the leading cause of dementia, affecting 50 million people worldwide [41]. Its neuropathological hallmarks include extracellular β-amyloid (Aβ) plaques and intracellular hyper-phosphorylated tau (p-τ) in neurofibrillary tangles, accompanied with synaptic and neuronal loss [40,41,42]. AD can be classified as (i) familial or early-onset AD (EOAD) or (ii) sporadic or late-onset AD (LOAD) [43]. Both share almost similar pathophysiology [44,45]. Three causative genes, including presenilin 1 (PSEN1) [46], presenilin 2 (PSEN2) [47], and amyloid precursor protein (APP) [48], are involved in the pathogenesis of EOAD in an autosomal-dominant trait [49,50]. LOAD, however, comprises most AD cases (>95%) where the greatest risk factor is advanced age [51], while the common genetic risk factor is an allelic variation in apolipoprotein E (Apo E) [52]. Recent large scale studies of AD genetics, employing genome-wide association studies (GWAS), whole exome sequencing (WES), and whole genome sequencing (WGS), have defined additional genes whose variants contribute to increased risk [53,54]. These include Clusterin (CLU), Sortilin-related receptor-1 (SORL1), ATP-binding cassette subfamily A member 7 (ABCA7), Bridging integrator 1 (BIN1), phosphatidylinositol binding clathrin assembly protein (PICALM), CD2 associated protein (CD2AP), Complement component (3b/4b) receptor 1 (CR1), CD33, triggering receptor expressed on myeloid cells 2 (TREM2), and phospholipase D3 (PLD3) [55,56,57]. Intriguingly, more than 50% of validated gene variants are implicated in innate immune and microglial functions [58,59,60], including the top two AD risk genes, APOE and TREM2 [61,62]. Epigenomic analysis shows that AD GWAS loci are preferentially enriched in enhancer sequences involved in innate immune processes [63,64] as well as endocytosis, cholesterol/sterol metabolism, and synaptic function [55,65,66]. TREM2 enhances the rate of phagocytosis in microglia and macrophages; modulates inflammatory signaling; and controls myeloid cell number, proliferation, and survival [67], and it has been revealed that triggering TREM2 receptor in microglial cells is closely associated with the pathogenesis of AD [68]. TREM2 modulates microglial functions in response to Aβ plaques and tau tangles [69,70]. In early AD, the absence of TREM2 leads to increased amyloid pathology that progressively becomes worse owing to the loss of phagocytic Aβ clearance [69]. In AD, TREM2 variants arise in part because of their reduced capacity to phagocytose Aβ [71].

While compelling evidence indicates that AD has a multifactorial etiology [72,73,74], neuroinflammation plays a central role in its etiopathogenesis [75,76], owing to its capacity to exacerbate Aβ and τ pathologies [77]. In vivo positron emission tomography (PET) studies provide direct evidence of increased microglia activation (inflammation) in the brains of AD patients [78,79,80]. The levels of pro-inflammatory cytokines in AD patient serum and post mortem brain are elevated [81,82], and Aβ can activate the brains’ innate immune cells [83,84]. The sustained inflammatory response in AD brains [85,86,87] extends beyond a reaction to neuronal loss [88] and involves microglia, astrocytes, oligodendrocytes, mast cells, cytokines, and chemokines, as well as complement [89]. These collectively play an integral role in the onset and progression of the disease [88,90,91]. Other early-onset processes involved in the etiology of the disease include mitochondrial dysfunction resulting in altered glucose metabolism [92] and oxidative stress [93]; chronic hypoperfusion [94]; and neuronal cell cycle re-entry that leads to neuronal tetraploidization (NT) [94], trisomy 21 mosaicism [95], and synapse loss [96]. These processes may synergistically interact to facilitate the neurodegenerative process in AD [93,97].

Here, we focus on recent progress made in understanding the role of inflammation in the etiopathogenesis of AD and describe a few immunomodulatory strategies being developed to selectively target the detrimental aspects of neuroinflammation without interfering with the defense mechanisms against pathogens and tissue damage.

## 2. Mediators of Neuroinflammation

### 2.1. Microglia

**Figure 1:**
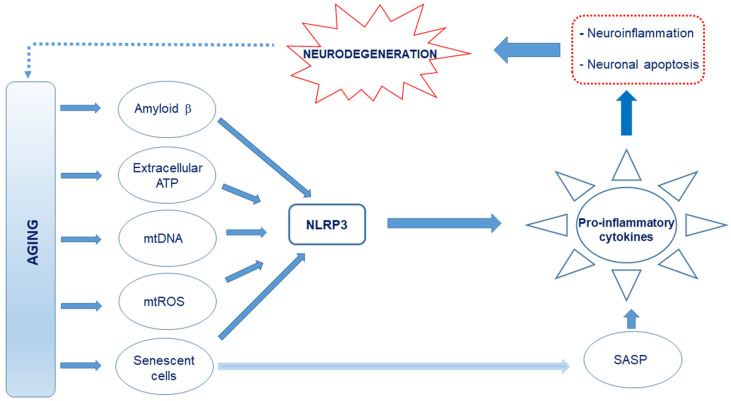
Schematic representation of the proposed causes of neuroinflammation in Alzheimer’s disease (AD). Age-related release of damage-associated molecular patterns (DAMPs) such as Aβ, extracellular ATP, and cell debris such as circulating mitochondrial DNA, which are capable of interacting with the Nod-like receptor 3 (NLRP3), creates an oxidative and neuroinflammatory environment through the excessive production and release of pro-inflammatory cytokines and reactive oxygen and nitrogen species (RONS). Further, mitochondrial reactive oxygen species (mtROS) and senescence-associated secretory phenotype (SASP) factors from the senescent cells, which also drive senescence in nearby cells, produce pro-inflammatory cytokines. This culminates in neuroinflammation and neuronal apoptosis.

Microglia are the CNS’s immune cells [98] and are different from peripheral and other tissue-resident macrophages [99,100,101,102,103,104]. They arise from yolk-sac fetal macrophages and are unique in their capacity for self-renewal [105,106,107]. Other tissue macrophages develop from precursors that emerge later in life [103,104]. They constantly survey their milieu and assess ongoing synaptic activity, mediate synaptic pruning, clear debris, and provide trophic support for neurons [108]. In pathological situations such as chronic stress, the blood brain barrier (BBB) may become compromised, allowing peripheral hematopoietic cells to cross into neural tissue and become part of the parenchymal microglia/macrophage pool [109,110]. In response to CNS insults such as neuronal injury or infection, microglia become activated to produce pro-inflammatory factors (M1 phenotype) or anti-inflammatory factors (M2 phenotype) [111]. An exquisite balance of anti-inflammatory mediators to heal and repair tissues and pro-inflammatory mediators to clear cellular debris and aggregated misfolded proteins is essential for the maintenance of a healthy CNS [27,111]. With advancing age, microglia acquire an activated phenotype and release pro-inflammatory cytokines such as IL-1β, TNF-α, and IL-6 [112,113,114]. In AD, microglia react to pathogen-associated molecular patterns (PAMPs) or danger-associated molecular patterns (DAMPs) [115,116,117] to assume a M1 phenotype, leading to an exacerbation of inflammation and an acceleration of disease progression [88] (Figure 1). Preliminary findings implicate a link between Aβ and neuroinflammation [118]. In AD brain slices, activated microglia surround both extracellular Aβ plaques and neurons containing neurofibrillary tangles (NFTs) [119,120]. It is thought that Aβ activate microglia, which then secrete IL1β, IL6, and TNFα, as well as (C-C motif) ligand (CCL 2/4/11), which lead to the recruitment of more microglia and astrocytes to the Aβ locus [121]. Microglia phagocytize Aβ through a range of cell surface receptors, including cluster of differentiation (CD)-14, toll-like receptor (TLR)-2, TLR4, α6β1 integrin, CD47, and scavenger receptors such as CD36 [122,123,124,125]. In AD, the accumulation of Aβ throughout the brain results partly from the failure of microglia to remove extracellular Aβ [126,127,128] and AD cortical specimens reveal that the microglia surrounding plaques have impaired Aβ uptake [127,129,130]. It has been shown in human and animal studies that inflammation influences APP processing overall [131,132]. Initially, microglial activation may serve to eliminate Aβ [126,133,134,135], but their chronic activation may amplify the amyloid cascade [133,136] and lead to neurotoxicity [137,138]. In rat intraventricular hemorrhage (IVH) model of AD, Aβ accumulation tracks with neuroinflammation and may contribute to the cognitive impairment [139].There is no consensus, however, regarding the relationship between in vivo microglial activation and Aβ plaque burden [140,141,142,143,144]. Aβ has recently been suggested to be an antimicrobial peptide that fibrilizes in order to activate the innate immune defense system and protect the host from a wide range of infectious agents [145].

### 2.2. Astrocytes

Astrocytes are the most abundant cell type in the CNS and a critical part of the tripartite synapse [146]. They are highly sensitive to their environment and rapidly respond to CNS needs and insults [147]. They also regulate the maturation of neurons and help maintain their function [147,148]. They are found in various states of activation and can be neuroprotective (reducing inflammation and stimulating repair) or neurotoxic (promoting inflammation that may result in neurodegeneration) [149,150]. They respond to inflammatory molecules such as cytokines and chemokines and are able to detect aggregated proteins such as Aβ [148,151,152]. They hypertrophy upon activation and upregulate glial fibrillary acidic protein (GFAP) expression [153,154]. Reactive astrocytes are a distinct trait of AD patient brains [155] and are also a feature of AD mouse model brains [156,157,158]. Intralaminar astrocytes are atrophied and severely disrupted in post-mortem AD brains [159]. In the 3xTg, which contain three mutations associated with a familial AD (APP Swedish, MAPT P301L, and PSEN1 M146V) mouse model, atrophic astrocytes appear in the entorhinal cortex (EC) as early as 30 days and are present until Aβ plaques begin to emerge at 12 months of age [160]. This phenomenon also occurs in other AD mouse models including the 5xTG-AD, PDAPP-J20, and Swiss 3 mice [161,162,163,164]. When astrocytes are created from familial and sporadic AD-induced pluripotent stem cells (iPSC), they have an atrophic phenotype in vitro [165]. Inhibiting astrogliosis in AD mouse brains results in Aβ accumulation with increased histopathology [166] and they are associated with cognition [167]. This may result in a breach of the blood brain barrier (BBB), leading to an infiltration of peripheral immune cells, aggravating neuroinflammation and inducing neurotoxicity by impairing glutamate homeostasis [168,169], and generating altered Ca^2+^ signaling [170].

### 2.3. Oligodendrocytes

The main function of oligodendrocytes is to provide support and insulation to the axons by forming myelin sheaths around nerve fibers. Their involvement in AD is not fully understood, although emerging evidence implicates their potential role in pathogenesis and progression of AD [171]. Tsai et al. recently reported that oligodendrocytes are severely impaired in AD [172] and, indeed, there is focal loss of oligodendrocytes and a reduction in myelin proteins near Aβ plaques [173,174,175]. Aβ not only impairs the survival and maturation of oligodendrocyte progenitor cells (OPCs), but also hampers the formation of the myelin sheath [176]. Neuroinflammation and oxidative stress may also contribute to oligodendrocyte dysfunction and death [175].

### 2.4. Myeloid Cells Other Than Microglia

The other monocytic cells found in the CNS include perivascular macrophages that line blood vessels of the brain, macrophages within the choroid plexus, and meningeal macrophages in the leptomeninges [177]. Dendritic cells, monocytes, and granulocytes are found in the meninges and are recruited to the brain during or after an insult or other pathology [178,179]. The CNS-resident macrophages express scavenger receptors (SR) and TLRs that facilitate phagocytosis and degradation of Aβ [180,181]. In SR knock out mouse models of AD, Aβ accumulates in the parenchyma and the animals have cognitive deficits [124,182]. In the CD11c-DNR mouse model of AD, which expresses a dominant-negative form of the TGF-β receptor under the control of the CD11c promoter, the brain levels of Aβ are reduced by up to 90%, the Aβ plaques and cerebral vasculature are surrounded by macrophages [183], and the behavior of the animals is significantly improved [183]. The migration of peripheral monocytes is dependent on C-C chemokine receptor type 2 (CCR2) [184]. Blocking transforming growth factor (TGF)-β signaling increases peripheral myeloid cell infiltration into the CNS and significantly reduces Aβ burden [183]. It is still not exactly clear how myeloid infiltration into the brain contributes to damage or clearance of pathological proteins.

## 3. Defective Autophagy and Neuroinflammation

Cells degrade protein aggregates and damaged organelles by autophagy and defective mitochondria by mitophagy [185,186,187,188]. With advancing age, autophagy gradually subsides and this decline is linked to defective mitochondria and results in inflammaging [189]. Damaged cellular and organelle components that accumulate as a result of inadequate autophagy are released as damage-associated molecular patterns (DAMPs) [190,191,192]. Dysfunctional mitochondria that are not eliminated by mitophagy release large amounts of mitochondrial DNA (mtDNA) into the cytosol and, together with ROS [193,194], metabolites such as ATP, fatty acids, Aβ, succinate, per-oxidized lipids, advanced glycation end-products, altered N-glycans, and HMGB1 are also recognized as DAMPs and trigger an innate immune inflammatory response [195,196] by directly activating TLR9. This initiates the transcription of pro-inflammatory cytokines such as IL-6, TNF-α, IL-1β, and MMP-8 [197] and activates the Nod-like receptor 3 (NLRP3) inflammasome, a key regulator of inflammation [198,199,200], to activate caspase-1 and facilitate IL-1β and IL-18 maturation as well as gasdermin D-mediated pyroptotic cell death [201,202,203]. These inflammatory responses can be blocked by Pro-IL-1β degradation in autophagosomes [204]. Further, the mitochondrial derived peptide (MDP) known as mitochondrial open reading frame of the 12S ribosomal RNA type-c (MOTS-c) [205] reduces inflammation by inhibiting cytokines such as TNF-α and IL-6, while simultaneously promoting an anti-inflammatory response. MOTS-c stimulates IL-10 as well as signal transducer and activators of transcription 3 (STAT3) and aryl hydrocarbon receptor (Ahr), which inhibit NFκB expression and proinflammatory cytokine production [206]. Another mitochondrial peptide, humanin, also has anti-inflammatory effects [207,208]. The chronic sterile low-grade inflammation elicited [4] may culminate in immunosenescence [209] and compromise neuronal function [32]. This may partly explain why dysregulated NLRP3 inflammasome activation is observed in AD [210,211]). Eliminating damaged and dysfunctional mitochondria by mitophagy may prevent the hyperinflammation triggered by NLRP3 inflammasome activation [212].

## 4. Mitochondrial Dysfunction and Immunometabolism

Mitochondrial dysfunction with decreased oxidative phosphorylation (OXPHOS) and increased glycolysis is observed in AD microglia [213]. ]. Microglia exhibit high metabolic flexibility to cope with their high energy demands [214]. Microglial activity becomes compromised with age as mitochondrial activity declines with age and especially in age-related diseases such as AD [215] and they have a low mitochondrial turnover [213,216]. Exposure to Aβ and tau activates a proinflammatory phenotype that is accompanied by a shift of the metabolic profile from OXPHOS to glycolysis. If the inflammatory process is prolonged, bioenergetic failure involving both glycolysis and OXPHOS occurs [217]. In the 5XFAD mouse model of AD, enhancing glycolytic metabolism restores microglial phagocytic activity [217]. Increased mitochondrial fragmentation is seen in cellular and animal models of AD and is associated with the increased pro-inflammatory cytokine production and neurotoxicity [213]. These mitochondrial perturbations in activated microglia are propagated to other cell types, including astrocytes and neurons, exacerbating the disease outcome [213]. Increased glycolysis and upregulated expression of glucose transporters as well as glycolytic enzymes in activated microglia are a well-established phenomenon [218,219]. Increased microglial glycolysis is often coupled with the increased secretion of proinflammatory cytokines, exacerbating neurotoxicity, and escalating ongoing neurodegeneration [219,220]. Glycolysis is upregulated in microglial cells during the early phase of AD, coupled with increased phagocytic activity followed by an “immune tolerant” phase, during which glycolysis and oxidative phosphorylation were both disrupted, with less phagocytic potential [217]. Microglial activities can be restored through increasing metabolic functions with interferon-gamma (IFN-γ), resulting in increased microglial clustering around Aβ plaques, phagocytosis, and TNF-α production. Conversely, in AD models, oxidative phosphorylation increased phagocytosis and migration and reduced proinflammatory cytokine production [221].

## 5. Oxidative Stress and Neuroinflammation

Neuroinflammation and oxidative stress are key pathologic signatures of AD [222,223,224,225]. RONS are produced by all aerobic cells, and while they are essential signaling molecules [226,227], a redox imbalance is detrimental and plays an important role in the inflammatory process in aging as well as in AD [226,228,229]. While cells of innate immunity produce copious amounts ROS within the central nervous system (CNS), it is not clear whether neuroinflammation induces oxidative stress or if it is the elevated levels of ROS that cause neuroinflammation [230,231], especially in a situation where there is already excess oxidant production in the face of age-associated weakened antioxidant defense [230,232].

Elevated RONS levels damage primary cellular components including lipids, proteins, and DNA [226]. Increased oxidative stress biomarkers (i.e., malondialdehyde (MDA), glutathione peroxidase (GSH-Px), and protein carbonyl (PC)) correlate with raised levels of inflammatory cytokines and both are associated with low cognitive performance in institutionalized elderly people [233]. Mitochondria are the most important source of intracellular ROS and mitochondrial dysfunction leads to significant ROS increase [226]. Alternative mechanisms that produce ROS include NADPH oxidase (NOX), immune activation, xanthine oxidase (XO), arachidonic acids (AA) metabolism, and so on [226,234]. mtDNA mutations that accumulate with age disrupt the mitochondrial respiratory chain and lead to excessive mitochondrial ROS (mtROS) production, which, in turn, accelerates the emergence of new mtDNA mutations, leading to cellular senescence, further aggravating the inflammaging process. A vicious oxi-inflamm-aging cycle is thus created in aging and exaggerated in AD [235,236].

## 6. Cellular Senescence and Neuroinflammation

Stimuli such as mitochondrial dysfunction, persistent DNA damage, and exposure to DAMPs can initiate cellular senescence [237,238]. Senescent cells accumulate with aging and secrete proinflammatory and matrix-degrading molecules as part of a senescence-associated secretory phenotype (SASP), which is linked to age-related tissue inflammation and disease [239]. Senescent cells are deleterious to non-senescent neighboring cells. They develop a SASP and secrete cytokines, chemokines, ROS, and proteases that create a noxious microenvironment [240,241,242] that promotes inflammaging [243]. Senescent neurons have been described in old rodent and human brains [244,244,245], suggesting a proliferation-independent senescence-like mechanism in post-mitotic cells including neurons [246]. This concept is described as “amitosenescence” and involves an attempt by post-mitotic cells (particularly neurons) to re-induced cell cycle [247,248]. SASP may account for several observed disease phenotypes in AD [249,250,251,252]. The SASP is a very heterogeneous phenotype [253] and the secreted components vary on the basis of cell type and triggering factors, but generally consist of interleukins (IL-1α, IL-1β, and IL-6), chemokines (IL-8 and growth-regulated-α protein), growth factors (fibroblast growth factor 2 and hepatocyte growth factor), metalloproteinases (interstitial collagenase (also known as MMP1), stromelysin 1 (also known as MMP3), and collagenase 3 (also known as MMP13)), as well as other insoluble proteins and extracellular matrix components [254,255,256]. Their effects are mainly paracrine, but may become systemic as some of the soluble mediators may get into the circulation [257,258,259]. Prominent SASP regulators include p38MAPK, NFκB C/EBPβ, GATA4, and mechanistic target of rapamycin (mTOR) [260,261,262]. Senescent markers are up-regulated in the astrocytes of AD patients and Aβ elicits senescence in astrocytes in vitro via ROS accompanied by p38, IL-6, and IL-8 up-regulation [263]. When rat astrocytes are cultured, they exhibit signs of senescence including intense SA-β-gal staining, elevated ROS production, and bioenergetic deficits. This compromises their ability to maintain neurons and adversely affects the aging brain [264,265]. Depleting human astrocytes of glutathione in vitro activates SASP-associated pathways (NF-kB and p38MAPK) and IL-6 secretion [266]. In vitro, both human and rodent astrocytes upon replicative or stress induced senescence become flattened and enlarged and adopt senescence-associated heterochromatin formation (SAHF) and have elevated levels of p53, p21CIP1, p16INK4a, and SA-β-gal [267,268]. Further, oligodendrocyte precursor cells (OPCs) in an AD mouse model show a proinflammatory phenotype along with increases in p16 and p21 expression near Aβ plaques, and Aβ can induce senescence in cultured OPCs [252]. Mouse models of Aβ plaque accumulation lend credence to the fact that OPCs localize to plaques. In the 3xTg-AD mice AD [269],158], hypertrophic OPCs infiltrate and accumulate around Aβ plaques [270] and in the TgAPP/PS1 mouse model, the OPCs surrounding Aβ plaques have increased Cdkn2a mRNA expression, and SA β-gal and IL-6 levels (SASP factor) reflect what is seen in the human brain. This suggests that extracellular protein accumulation may negatively impact OPC function [252]. While IL-6 and TGF-β mRNA are upregulated in AD patients [271,272], an inflammatory response from resident immune cells is also prominent in AD [88]. Generally, senescent cells are cleared by natural killer cells (NKs) [273,274,275] and macrophages [276,277,278]. The CNS being under relative immune privilege, T-cells, NKs, and peripheral macrophages normally have limited access to the meninges and choroid plexus and far-limited access to the CNS parenchyma [279,280,281]. However, this immune privilege is not absolute, and cells of the CNS are sensitive to inflammatory events occurring both within and outside of the brain [282]. In the aging brain and especially in the AD brain, the BBB is compromised, leading to peripheral immune cell infiltration [283,284,284,285,286]. This allows the infiltration of peripheral immune cells [287] and possibly infectious pathogens. Although microglia are the CNS resident macrophages, there is no evidence that they selectively kill senescent cells [261]. Getting rid of CNS senescent cells would require infiltrating CD4^+^ T-cells, as peripheral macrophages appear to depend on these cells to kill senescent cells outside the CNS [277]. As the clearance of senescent cells in the CNS is limited in non-aged healthy individuals, senescent cells, SASP, and secondary senescence may continue in the brain for several years or even decades.

## 7. Meta-Inflammation (Metaflammation)

Late life obesity/metabolic disease and diabetes (T2DM) contribute to low-grade non-resolving inflammation and neuroinflammation [91,288] and increase the risk of developing AD [289]. AD has been referred to as “type 3 diabetes” [290,291]. Adipose tissue inflammation in obesity is sterile, chronic, and low grade and affects the metabolic control of nutrient flow in adipose tissue, liver, muscle, and pancreas by inducing insulin resistance, and it differs from the typical inflammatory response to pathogens [292,293,294,295]. Indeed, peripheral insulin resistance leads to decreased insulin signaling in CNS that is associated with an alteration in brain metabolism and activation of inflammatory pathways [296]. Hyperglycemia in T2DM increases AD risk by exacerbating microglia and astrocyte-mediated neuroinflammation and neuronal injury [297]. Individuals with advanced age, obesity, T2DM, or hypercholesterolemia are more likely to be affected by AD [298,299].

## 8. Exercise and Inflammation

Endurance exercise (EE) has anti-inflammatory properties that can reduce the risk of several metabolic disorders [300,301,302]. Some of these effects arise from the IL-6 secreted from skeletal muscles into circulation in response to EE [303] and include the acute phase of immune response [304] as well as glucose and lipid metabolism [305,306], and have been shown to reduce the risk and severity of many chronic diseases, and the benefits extend to the brain [302]. One of the long-term immunometabolic adaptations is mediated by energetic stress, which induces beneficial molecular adaptations in adipose tissue and immune cells [307]. In the brain, this results in the upregulation of IL-10 that activates mouse microglia and astrocytes [308,309]. The modulatory effects of exercise on inflammation both centrally and peripherally have a protective effect on cognitive function and may be beneficial to patients with AD [310,311], as it elevates levels of circulating growth factors (such as insulin-like growth factor 1, IGF-1) and neurotrophins (such as the brain-derived neurotrophic factor, BDNF) [310,312].

## 9. Gut Microbiota and Inflammation

The gut microbiota is less diverse with age and contains more Bacteroides, compared with the higher presence of Firmicutes in younger adults [313,314,315]. There is also a correlation between microbial diversity, frailty scores, and environmental factors such as dietary pattern in elderly individuals [313,316]. These changes can initiate dysbiosis, and the prevalence of pathogenic species in the intestinal microbial composition results in elevated levels of systemic proinflammatory markers (IL-6, IL-8, TNF-α, and CRP) [313,316] associated with the pathogenesis of AD [317,318]. The microbiota can also modulate events in the brain via vagus nerve activation, neuropeptide and neurotransmitter release, short-chain fatty acids (SCFA), α-amino-β-methylaminopropionic acid (BMAA), and lipocalin-2 release [319,320,321]. These signals reach the brain and influence the microglial maturation and activation [322], facilitating immune surveillance, regulation of hypothalamic-pituitary-adrenal (HPA) axis, synaptic pruning, and clearance of debris [323]. Further, neurobehavioral complications associated with peripheral infections may be facilitated by advancing age because hippocampal processing is more easily disrupted when the peripheral innate immune system is stimulated in older animals [324,325].

## 10. Complement in the Brain

While complement proteins are mainly synthesized in the liver, complement proteins and their cognate receptors and regulators are expressed throughout the CNS [326,327,328,329]. The intact blood–brain barrier (BBB) restricts and prevents access of complement proteins from the periphery. Local production is, therefore, particularly important for innate immune defense in the healthy brain. Even in the healthy brain, however, there are regions where the BBB is compromised, particularly in the aged normal brain where evidence of barrier loss in and around the hippocampus has been described [330], and this is more severe in the AD brain [327]. In the post-mortem AD brain, complement protein and activation products surround plaques and tangles [326]. Numerous mediators are implicated, including ROS and activation of tissue metalloproteinases, but the dominant pathway to BBB breakdown is inflammation, either central or systemic [331,332]. BBB impairment in AD may be much more subtle, localized to areas of pathology, and affecting specific transport processes—e.g., the transport of Aβ [284,333]. C3a/C3aR signaling via intracellular Ca^2+^ mediates vascular endothelial cadherin junction and barrier integrity in an in vitro model of the BBB. Microglial reactivity can be inhibited by inactivation of C3aR1, and this restores hippocampal and cortical volumes in aged brains, suggesting an association between impaired BBB, inflammation, and neurodegeneration [334,335]. Complement plays complex roles in brain homeostasis and likely has both protective and exacerbating effects on disease [327]. Evidence suggests that complement restricts Aβ plaque formation and aids clearance of plaque components, but also contributes to the switch of microglia and astrocytes into activated neurotoxic cells that drive the pathology [336,337,338,339].

## 11. Possible Intervention for Neuroinflammation in AD

In chronic inflammation, the initial response is not adequately resolved, leading to the accumulation of TNF, IFNs, and IL-6 as well as immune cells, apoptotic cells, and debris at the site of insult/injury, compromising functional homeostasis [340]. While there are concerted efforts to develop compounds that target specific facets of inflammation, viable strategies should seek to resolve neuroinflammation without impacting the defense mechanisms against pathogens and tissue damage. We depict a few immunomodulatory strategies being developed to delay the onset and/or progression of AD.

### 11.1. Targeting TNF-α

TNF-α plays a central role in the initiation and maintenance of the inflammatory response, and effects of TNF-α in the CNS can be either homeostatic or pathophysiological [341,342]. It regulates synaptic plasticity, microglial activation, astrocyte-induced synaptic strengthening, and glutamatergic transmission in the healthy CNS [343,344], while in pathological conditions, the copious amounts of TNF-α released by microglia mediate a chronic inflammation that leads to neuronal dysfunction and cognitive impairment [344], as seen in transgenic mouse models of AD [345]. 5XFAD/Tg197 AD/TNF double-transgenic mice develop a human TNF-α (huTNF-α) expression induced Aβ plaques deposition and arthritis. Systemic treatment with infliximab, an anti-huTNF-α antibody that does not cross the BBB, protects neurons, reduces gliosis, and prevents infiltration of peripheral immune cells without altering brain huTNF-α levels [346,347]. In AD patients, TNF-α not only co-localizes with Aβ plaques in the brain, but the levels are also elevated in the plasma and cerebrospinal fluid (CSF) and correlate with the severity of the disease [348]. TNF-α specific monoclonal antibodies such as infliximab, adalimumab, and the recombinant fusion proteins etanercept have been developed for peripheral inflammatory conditions and have been demonstrated to be effective and well tolerated in patients with a wide range of inflammatory conditions [349]. These drugs have been tested on AD rodent models via central and peripheral routes of administration [345,350]. Adalimumab significantly attenuated neuronal damage and neuroinflammation, decreased beta secretase-1 protein expression and Aβ1-40 plaques, and improved cognitive functions in Aβ1-40-injected mice [351,352], suggesting possible clinically meaningful outcomes in patients with AD. Indeed, patients with rheumatic disorders that are treated with TNF-blocking agents have a lower risk for developing AD [353]. XPro1595, a second-generation TNF-α inhibitor, targeting only the soluble form of TNF-α, thus preserving the neuroprotective transmembrane TNF-α signaling pathways [354], has been evaluated in three different mouse models of AD. Peripherally administered, XPro1595 reduces brain amyloid deposition and age-dependent increase in activated immune cells and improves synaptic function [355,356]. Locally administered, XPro1595 reduced pre-plaque Aβ pathology in 3×TgAD mice [357] with concomitant reduction in microglia activation and improvement of synaptic and cognitive functions in aging rats [358]. While new TNF-α inhibitors that are able to cross BBB are being developed [359], the use of the above compounds is limited to peripheral targeting of TNF-α as they are not able to readily cross the BBB [345].

An alternative approach under consideration for AD is the development of inhibitors of TNF-α synthesis [343]. Thalidomide and its derivatives, also referred to as immunomodulatory imide drugs (IMiDs), target the 3′-untranslated region (3′-UTR) of TNF-α mRNA, inhibiting TNF-α cytokine production. Compared with similar anti-inflammatory drugs, currently marketed IMiDs have improved BBB permeability and bioavailability, making them viable candidates for neurological disorders [360]. Chronic thalidomide administration significantly reduces both astrocytes and microglia activation, and Aβ generation in brains of APP23 transgenic mice through inhibition of β-secretase (BACE1) [361,362]. 3,6′-dithiothalidomide (3,6′-DT) effectively lowers TNF-α, nitrite, and secreted amyloid precursor protein (sAPP) levels in vitro in LPS-activated macrophage-like cells. It also significantly reduces central and systemic TNF-α production and neuroinflammatory markers and restores hippocampal neuronal plasticity in LPS-challenged rats [363,364]. Chronic 3,6′-DT administration ameliorates Aβ-induced neuroinflammation and microglial activation, preventing neurodegeneration and improving memory in an AD mouse model of stereotaxic intracerebroventricular Aβ1-42 [365] and reduces multiple hallmark features of AD, including glia activation, phosphorylated tau protein, APP, Aβ peptide, and Aβ-plaque number along cognitive dysfunction in 3×Tg-AD mice, and leads to synaptic preservation [363,366]. When treated with 3,6′-DT from 4 months of age, these mice showed only Aβ deposition within neurons, but no plaque or τ pathology is evident. This is associated with an increase in TNF-α levels and improved working memory performance [363, 366]. These promising preclinical studies using IMiDs indicate their potential of progressing from the bench to clinical trials and, eventually, to the bedside of AD patients.

### 11.2. Senolytics

Senescent cells secrete proinflammatory cytokines, chemokines, and tissue-damaging proteases that negatively impact their surrounding microenvironment and accelerate aging and age-related diseases [367]. Senolytics are drugs that selectively eliminate senescent cells [368,369] and apparently provide beneficial effects in rodent models of aging [370], although a causal link between senescent cell accumulation and AD is yet to be established. Dasatinib, a tyrosine kinase inhibitor with BBB penetrance, decreases microgliosis and is neuroprotective in preclinical models of AD [371,372]. Quercetin is a naturally occurring flavonoid that ameliorates AD pathology and protects cognitive and emotional function in aged triple transgenic AD disease model mice [373]. In combination, Dasatinib and Quercetin (D + Q) have favorable side effect profiles, which makes them attractive for in-human trials [368,374,375]. Fisetin (F) is flavonoid senolytic occurring in many plants, fruits, and vegetables that is widely available as a nutritional supplement [369,376]. In old mice, intermittent F administration enhanced cognitive function [375]. Intermittent administration of senolytics into a τ^+^ mouse line (rTg(tauP301L)4510) with mild τ overexpression that exhibit neurocognitive symptoms only after 22 months (equivalent to 70 human years) decreases brain senescent cells, neuroinflammation, and gliosis; enhances neuron density; partially restores lost cortical brain tissue; and decreases ventricular enlargement [250]. These changes are associated with the clearing of 35% of proinflammatory senescent NFT-containing neurons. Zhang et al. have demonstrated that D + Q clear brain senescent oligodendrocytes, decrease neuroinflammation, and improve memory acquisition and retention [252]. Senolytics are selective for senescent, but not normal oligodendrocyte lineage cells derived from human brains as well as in the transgenic mice [375]. In AD patient brains and brains of rTg4510 mice, only Olig2 and NG2 expressing OPCs that are associated with Aβ plaques exhibit a senescence like phenotype, while astrocytes, microglia, and oligodendrocytes that are exposed to Aβ do not. Treating these mice with senolytics selectively eliminates senescent cells, ameliorates inflammation and cognitive decline, and reduces Aβ accumulation [252,377].

### 11.3. Targeting the Inflammasome

Targeting the NLRP3 inflammasome is gaining traction as a therapeutic strategy in inflammatory diseases [378,379]. Several approaches endeavor to inhibit the activation of the NLRP3 inflammasome and reduce microglial cytokine production [380]. The NLRP3 inflammasome significantly contributes to neuroinflammation and age-related cognitive decline and is potently activated by Aβ [381]. Novel specific inhibitors of the NLRP3 inflammasome are currently in pre-clinical or clinical trials [382] (ifmthera.com/pipeline). MCC950 (also known as CP-456,773 and CRID-3), a potent inhibitor of NLRP3, promotes microglial clearance of Aβ, reduces Aβ accumulation, and improves cognitive function in APP/PS1 mice [383,384,385,386]. Both MCC950 and Inzomelid, another potent, selective, brain-penetrant NLRP3 inflammasome inhibitor, are expected to move into Phase II trials for a range of disorders, including Parkinson’s, Alzheimer’s and, motor neurone disease (https://www.europeanpharmaceuticalreview.com/news/111201/patents-for-nlrp3-inflammasome-inhibiting-compounds-granted-in-us-and-europe/. Accessed on 20 March 2021).

### 11.4. Targeting Immune Checkpoints

The PD-1 (programmed cell death-1) receptor (an inhibitory immune checkpoint receptor (ICR)) is expressed on the surface of activated T cells. Together with its widely expressed ligand PD-L1, PD-1 plays an important role in maintaining immune homeostasis and self-tolerance [387,388]. Persistent antigen stimulation and inflammation in pathological situations increase T cell surface expression levels of ICRs [389]. This leads to their interaction with their ligands on antigen presenting cells (APCs) that “exhausts” the T cells, inducing a hypofunctional state [390,391]. This mechanism could be manipulated to either quench or enhance the immune response as a therapeutic target and depends on recruited immunosuppressive regulatory T cells (Treg) [392]. This anti-PD-1/PD-L1 based immunotherapy has been effectively utilized for many cancers [393], and is now also a therapeutic strategy in AD animal models to induce a robust immune response against neurotoxic proteins [394]. Indeed, immunoneutralization of tumor necrosis factor-related apoptosis inducing ligand (TRAIL), a modulator of Treg cell functions, reduces neuroinflammation in a mouse model of AD [395]. In AD mouse models with advanced amyloid pathology, using PD-1 immune checkpoint blockers to overcome immune tolerance induces a robust systemic IFN-γ response with subsequent recruitment of monocyte-derived macrophages (MΦ) to the brain. This results in cerebral clearance of Aβ and improved cognition [396,397]. Altering the brain immunological environment by inhibiting the PD-1/PD-L1 axis in a mouse model of tau pathology mitigates cognitive deficits and cerebral pathology [398]. Supporting these findings, it has been shown that functional PD-1 is expressed in hippocampal neurons and anti-PD-1 treatment rescues synaptic transmission and plasticity and potentiates learning and memory [388]. In the 5XFAD AD mouse model and DM-hTAU mouse model of frontotemporal dementia, neuroinflammation and cognitive deficits are shown [398]. Although the immune checkpoint blockade based-therapy represents a promising therapeutic strategy for AD and age-related dementia, further research is needed before PD-1/PD-L1 based clinical trials are conceived for these disorders. In fact, it is important to note that several PD-1 antibody inhibitors developed for other indications activate the peripheral immune system, but fail to modulate monocyte-derived macrophage infiltration and progression of brain Aβ pathology in three different models of AD [399,400]. A different study using the same PD-1 checkpoint blockade [396,398] reported only a modest improvement of locomotor activity without any effect on cognition or tau pathology in a transgenic AD model [401]. PD-1 inhibitors may be able to reduce plaque load and improve cognition [396].

### 11.5. Targeting CD38

Daratumumab, a first-in-class humanized monoclonal antibody against the CD38 epitope, was approved and indicated for multiple myeloma patients refractory to conventional therapy [402]. CD38 is a NAD glycohydrolase that is expressed by neurons, astrocytes, and microglial cells and plays an important role in neuroinflammatory and brain repair processes [403]. Its role in AD was evaluated and it was found that deleting CD38 significantly reduces both soluble Aβ and plaque levels and improves spatial learning in mice [404]. In an APPswePS1ΔE9 mouse model of AD, loss of CD38 results in significant reduction of Aβ plaques and an improvement of learning performance. In these APPswePS1ΔE9.CD38-/- mice, both β- and γ-secretase activities are reduced, suggesting that the loss of CD38 is neuroprotective in this AD model [404]. So, while there is still no direct evidence implicating CD38 in NDDs, it might be a novel therapeutic target for modulating Aβ production and neuroinflammation in AD.

### 11.6. Targeting CD33

CD33 has been identified by GWAS among the leading risk factors for AD [405]. In the brain, it is exclusively expressed by microglia and infiltrating macrophages [406,407], and it has been shown in CD33–/– mouse models that knocking out CD33 reduces Aβ levels and amyloid plaque load in the brain [407]. As APP processing is not altered in these animals, the phenotype likely results from a more efficient phagocytic clearance of Aβ. Other studies have shown that overexpression of ectopic CD33 in microglial line BV-2 cells reduces Aβ uptake [407]. Indeed, subtype-selective sialic acid mimetic called P22 binds to CD33 and increases uptake of the toxic Aβ into microglial cells [408], making the sialic acid-binding site on CD33 an attractive pharmacophore for developing therapeutics that promote clearance of the Aβ.

### 11.7. Targeting IL-12/IL23

Inhibition of the IL-12/IL-23 pathway by genetic ablation or pharmacological manipulation significantly reduces cerebral Aβ load and cognitive deficit [409]. IL-12 and IL-23 subunit p40 production by microglia is increased in the APPPS1 AD mouse model. Genetic ablation of the IL-12/IL-23 signaling molecules p40, p35, or p19 reduces cerebral Aβ load and the biggest effect comes from ablation of p40 or its receptor complex [409].

Moreover, immunizing of AD transgenic mouse models with Aβ42 prevents Aβ plaque formation along with decreased expression of the IL-12Rβ1 receptor in T cells [409,410]. Co-blockade of IL-12 and IL-23 via targeting of p40 reduces Aβ burden [409,411,412]. Indeed, antibodies against p40, IL-12, or IL-23 (European patent EP2661445A2) have been developed for the prevention or treatment of AD.

### 11.8. Targeting Th1 Response

Glatiramer acetate (GA) (Copaxone), a synthetic analog of myelin basic protein [413], the first disease-modifying drug approved for treatment of multiple sclerosis (MS) [414] can be used to safely boost T-cell responses without the risk of autoimmune disease, as it weakly cross-reacts with myelin-derived autoantigens [415]. It is thought to mediate its effects by stimulating Th2 response, possibly by suppressing the inflammatory Th1 response [416], increasing the frequency and function of Treg cells, modulating CD8+ T cells, and exerting an immunomodulatory effect on B cells [417]. In APPSWE/PS1dE9 double-transgenic, immunization with GA results in cerebral recruitment of pro-healing, highly phagocytic monocytes (Mo) and MΦ, greatly alleviating cerebral Aβ burden, reducing microgliosis and astrocytosis, resulting in improved hippocampal-based cognitive functions [418]. Further, T cell-based vaccination with GA in the same animal model of AD leads to enhanced neurotrophic support and hippocampal neurogenesis [419]. In APP-Tg mice, nasal vaccination with a proteosome-based adjuvant plus GA activates pro-healing microglia and robustly reduces Aβ fibrils levels [420]. GA-stimulated MΦ protect neurons from Aβ-mediated synaptotoxicity both in vivo and in vitro [401] and enhances cerebral recruitment of Mo-derived MΦ and reversing loss of cortical and hippocampal excitatory synapses in mouse models of AD. GA immunization enhances the expression of hippocampal early growth response protein 1 (Egr1), a protein negatively correlated with hippocampal Aβ plaque burden [421]. GA-based vaccination could provide a new viable avenue for immune therapy for AD.

### 11.9. Targeting Microglial P2Y6 Receptor

GC021109 is a small molecule that specifically targets microglial purinergic P2Y6 receptors, promoting their phagocytic activity and inhibiting the release of pro-inflammatory cytokines. It has been shown to be safe in a phase 1 trial of AD (www.alzforum.org/therapeutics/gc-021109;www.clinicaltrials.gov.ct2/show/study/NCT02386306) Accessed on 20 March 2021).

### 11.10. Cyclosporine and Tacrolimus

Cyclosporine and Tacrolimus are inhibitors of calcineurin and are used as immunosuppressive agents to prevent post-transplant organ rejection and for the treat autoimmune diseases and tuberous sclerosis tumors [422]. They have been shown to downregulate APP mRNA and protein expression protein in primary cultures of neonatal rat astrocytes [423]. Tacrolimus significantly quenches Aβ- and LPS-stimulated secretion of pro-inflammatory cytokines and increases microglial uptake of fibrillar Aβ in vitro. It also reduces spleen cytokine levels, microgliosis, and Aβ plaque burden in APP/PS1 mice [424] and ameliorates dendritic spine density deficits in plaque-bearing AD model mice [425]. When APP/PS1 AD transgenic mice were treated with FK506, microgliosis, cytokine levels, and Aβ plaque load were significantly reduced [424]. In Streptozotocin (STZ)-treated mice, Cyclosporine and Tacrolimus significantly reduce biochemical and histopathological alterations and age-related memory deficits, demonstrating their potential as therapeutic agents in cognitive dysfunctions, probably owing to their anti-amyloid, anti-oxidative, and anti-inflammatory properties [426,426].

### 11.11. Targeting p38MAPK

Another novel therapeutic agent in the pipeline includes the orally active p38 MAPKα inhibitor Neflamapimod [VX-745] that is being developed as a disease modifying drug for AD [427], with phase 3 trials slated for late 2021. It is able to reduce IL-1β levels (NCT02423200) [428] and improve patients’ attention and executive function.

### 11.12. CNS Targeting Anti-Complement Agents

In vivo tests in Tg2576 that overexpress the human amyloid precursor protein (hAPP) 695 isoform that harbors the Swedish double mutation found in some cases of familial AD, and 3xTg, which contain three mutations associated with familial Alzheimer’s disease (APP Swedish, MAPT P301L, and PSEN1 M146V) mouse models of AD, have shown that the cyclic hexapeptide PMX205 that potently and noncompetitively inhibits complement C5a Receptor 1 (C5aR1) decreases Aβ and τ deposits, reduces activated glia, and improves cognition, while not affecting the levels and physiological functions of C1q and C3 [429]. ANX-M1/ANX005, a humanized immunoglobulin G4 recombinant antibody against C1q developed by Annexon Biosciences, is safe at high doses (200 mg/kg) and is neuroprotective, preventing synaptic loss when Aβ fibrils are injected into the lateral ventricles [430]. It has proceeded to clinical trials [431]. C3aR antagonist SB290157 could decrease amyloid load and microgliosis [432]. It is important to recognize that complement may be neuroprotective early in the disease process by clearing debris, meaning that an intimate understanding of the therapeutic window where complement inhibition is most effect is crucial [326].

### 11.13. Novel Non-Steroidal Anti-Inflammatory Derivatives

CSP-1103 (also known as CHF 5074 or Itanapraced) is a non-steroidal anti-inflammatory derivative lacking cyclooxygenase inhibitory activity. It is a microglia modulator that has the potential to inhibit Aβ plaque deposition, reduce tau pathology, restore normal microglial function by increasing phagocytosis, and decrease production of pro-inflammatory cytokines [433]. It is currently in phase III clinical trials.

### 11.14. Calorie Restriction (CR)

CR has been shown to improve lipid and glucose metabolism, quench inflammation, and improve cardiovascular health [434]. This is thought to be via key nutrient and stress-responsive metabolic signaling pathways including IIS/FOXO, TOR, AMPK, Sirtuins, NRF2, and autophagy [435] that additionally work towards lifespan extension by CR [434]. Ultimately, the age-related proinflammatory upregulation of NFκB, IL-β, IL-6, TNF-α, and ROS is attenuated [436,437] and the beneficial effects include enhanced cognitive response [437].

### 11.15. Metformin

The biguanide drug metformin is a hypoglycemic drug widely prescribed to treat T2DM and metabolic syndrome [438,439]. Its mode of action is to simulate CR, and it increases lifespan and limits the onset of age-associated diseases across species [440,441]. It activates AMP-activated kinase (AMPK) [442], enhances autophagy and mitochondrial function, and quenches inflammaging [443].

### 11.16. Endurance Exercise

Endurance exercise (EE) is neuroprotective against AD [444,445]. Exercise activates continuous oxidative stress that induces a series of counteractive mechanisms. These enhance mitochondrial function and mitigate ROS-induced neurotoxicity, i.e., mitohormesis [446,447], and this is especially important in the hippocampus, which is particularly sensitive to oxidative stress [448]. EE also activates anti-inflammatory pathways [449,450].

### 11.17. Melatonin

N-acetyl-5-methoxytryptamine (Melatonin), which is synthesized from tryptophan, is able to activate both proinflammatory pathways. It is also capable of suppressing proinflammatory processes including NO release, activation of cyclooxygenase-2, NLRP3, gasdermin D, TLR-4 and mTOR signaling, cytokine release by SASP, and Aβ toxicity under different conditions [451,452]. In addition, it activates SIRT1 and upregulates Nrf2 while quenching NFκB activity and the release of IL-4 and IL-10, thus shifting microglia polarization towards an M2 phenotype [452].

### 11.18. Resveratrol

Resveratrol has been shown to affect aging and lifespan in mammals [453]. It is a potent natural SIRT1 activator that helps prevent aging-related decline in heart function and neuronal loss [454]. Resveratrol can also attenuate the phosphorylation of the mammalian target of rapamycin (mTOR) and S6 ribosomal protein (S6RP) while ameliorating inflammation [455]. Several cellular and animal studies show that SIRT1 is neuroprotective in neuroinflammation and neurodegenerative diseases. There are, however, conflicting results on the effects of resveratrol on AD subjects [456]. In one study, AD subjects who received resveratrol for 52 weeks had a significant reduction in the expression of cerebrospinal fluid matrix metallopeptidase 9 (MMP-9) and inflammatory markers [457], but in another clinical trial of AD, resveratrol had no beneficial effects [458]. A 26-week resveratrol treatment of healthy older adults improved memory performance and hippocampal functional connectivity [459]. These findings suggest that SIRT1 may be the potential target treatment of neuroinflammation and neurodegenerative disorders [460].

### 11.19. Antioxidants

Given the close association between oxidative stress and inflammaging [461] [461,462], targeting detrimental ROS at the production stage without affecting ROS signaling may improve immune function and ameliorate neuroinflammation [463]. Mitochondria-targeted antioxidants potently sequester reactive oxygen intermediates (ROIs) and confer greater protection against oxidative damage in the mitochondria than untargeted cellular antioxidants [464,465]. These mitochondria-targeted antioxidants such as (10-(6′-plastoquinonyl) decyltriphenyl-phosphonium) (SkQ1), MitoQ, MitoTEMPOL, and MitoVitE prevent apoptosis by mitigating the oxidative damage more effectively than untargeted antioxidants such as 6-hydroxy-2,5,7,8-tetramethylchroman-2-carboxylic acid (Trolox) [466]. Other such antioxidants include the following: 4,5-dihydroxybenzene-1,3-disulfonate (Tiron), which accumulate within the mitochondria by permeabilizing the mitochondrial membrane [467] and astaxanthin, a mitochondrion-permeable antioxidant, that can penetrate the blood–brain barrier and is effective in preventing and treating macular degeneration [468,469].

### 11.20. Probiotics

Gut microbiota are altered in the elderly; therefore, supplementation with specific strains of *Lactobacilli* and *Bifidobacteria* along with fructooligosaccharides and galactooligosaccharides may be beneficial [470,471,472]. They have been shown to attenuate inflammaging by down regulating IFN-γ and TNF-α and upregulating IL-10 [473,474]. Alteration of the gut microbiota can induce changes in brain activity, which raise the possibility of therapeutic manipulation of the microbiome in AD and other neurological disorders. This field of research is currently undergoing great development, but therapeutic applications are still far away [475]. The gut flora of cognitively impaired individuals and those with brain amyloidosis have an increased abundance of the pro-inflammatory species *Escherichia/Shigella*, while the abundance of anti-inflammatory species *E. Rectale* is reduced [476]. Fecal samples from elderly AD patients with AD induce a lower expression level of p-glycoprotein (a key mediator of intestinal homeostasis) in intestinal epithelial cells in vitro. p-glycoprotein dysregulation leads to inflammatory disorders of the intestine. Altered gut microbiota with disrupted intestinal homeostasis and induced inflammation may lead to neurodegenerative diseases like AD [476] via the gut–brain axis pathway [477]. Thus, remodeling the gut microbiota may be a novel therapeutic strategy for AD [473,474,478,479]. Altering gut microbiota through probiotics is a potential therapeutic strategy in AD [480,481,482,483].

### 11.21. Nutraceuticals

The nutraceutical NT-020, which is a proprietary blend of blueberries, green tea, vitamin D3, and carnosine, has been shown to reduce inflammation in elderly rats and prevent age-related cognitive decline and enhance neurogenesis [484,485,486,487,488]. Treating aged rats with NT-020 increases neurogenesis and serum from these animals’ rescued aged hippocampal neural stem/precursor cells (NPCs) and bone-marrow derived mesenchymal stem cells (MSCs) from age-related reduction in cell proliferation as measured by the 3-(4,5-dimethylthiazol-2-yl)-2,5-diphenyltetrazolium bromide (MTT) and 5-bromo-2′-deoxyuridine (BrdU) assays and serum from aged rats treated with NT-020 was not different from serum.

### 11.22. Essential Vitamins and Minerals

Vitamins and minerals play major roles in boosting the immune system to protect against certain infections and inflammation [489,490,491,492]. Vitamin E supplementation enhances IL-2 production and induces naive T cell proliferation [493,494,495]. Vitamin C is a potent water-soluble antioxidant and plays an important role in maintaining redox homeostasis within cells and is protective against ROS released by phagocytes [496,497,498,499]; therefore, it modulates the pro-inflammatory signaling pathways [500,501]. It also augments humoral response and cell-mediated immunity [500]. By accumulating within phagocytic cells, it enhances chemotaxis and phagocytosis [502] and is essential for apoptosis and clearance from the site of infection of spent neutrophils by macrophages to decrease potential tissue damage. Vitamin C also reduces inflammation by blocking the synthesis of TNF, IL-6, and IL-1β [502], and has been shown in vitro to promote IgG and IgM production [503]. Zinc (Zn) plays an important role as a structural and regulatory catalyst ion for several enzymes and transcription factors, and its deficiency results in significant decline in both the innate and adaptive immune responses and promotes systemic inflammation [504,505,506]. Zn supplementation is associated with lower TNF-α levels and reduced oxidative stress as well as lower incidents of infection [507,508,509]. Zinc deficiency activates the NFκB pathway and release of IL-2, IL-6, and TNF-α pro-inflammatory cytokines both in vivo and in vitro [A]. Zinc supplementation elicits inducible regulatory T cells production [510] and decreases ROS production [511].

### 11.23. Flavonoids from Epimedium and Icariin

Xia et al. have demonstrated that, in aged rats, Epimedium flavonoids and icariin attenuate the proinflammatory response while enhancing an anti-inflammatory response in inter alia the hippocampus, hypothalamus, and hypophysis, and mitigate neuroinflammation in aging [512,513]. Icariin acts via the AMPK/mTOR/ULK1 pathway to increase neuronal autophagy and enhance brain function in aged Sprague–Dawley (SD) rats [514]. Moreover, in the bilateral common carotid arteries occlusion (BCCAO) rat model of AD, which manifests as neuronal morphological damage along with Aβ deposition in the hippocampus and cognitive deficits of Aβ in rat hippocampus, oral administration of icariin reduces hippocampal expression of APP and BACE1, and induces the expressions of insulin-degrading enzyme (IDE) and a disintegrin and metalloproteinase domain 10 (ADAM10). This is accompanied by a decrease in TGF-β_1_ signaling via inhibition of Smad2/3 phosphorylation [515]. Icariin also inhibits LPS induced IL-s and TNFa production while increasing expression of NFκB p-p65 and (TLR)4–myeloid differentiation factor 2 (TLR4/MD-2) complex [516]. Tea flowers extract (TFE) has been shown to enhance p-AMPK and SIRT1 expression while reducing acetyl-NFκBp65 expression, and this is associated with a downregulation of IL-1β,TNF-α levels, inhibiting the pro-inflammatory response through the AMPK/SIRT1/NFκB pathway in aging rats [517].

## 12. Conclusions

**Figure 2:**
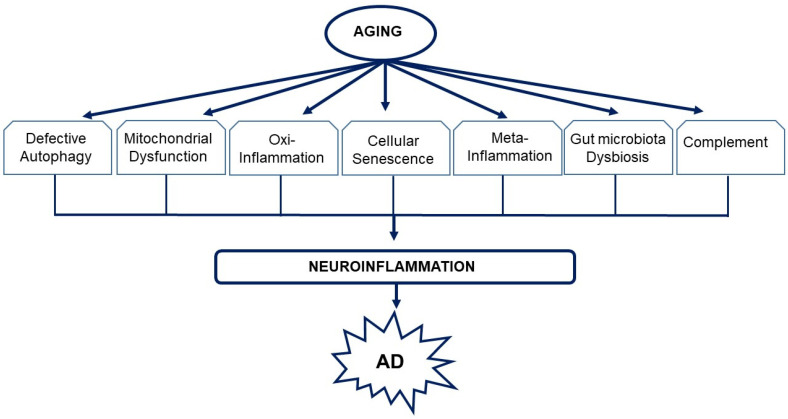
The interactions between aging and neuroinflammation in the pathogenesis of AD. With age, several cellular and molecular mechanisms elicit chronic sterile low-grade inflammation. This interaction of exogenous and endogenous risk stimuli, including defective autophagy, mitochondrial dysfunction, oxi-inflammation, cellular senescence, meta-inflammation, gut microbiota dysbiosis, and complement, triggers neuroinflammation. The resulting inflammatory mediator secreted drives the pathophysiological mechanisms of AD.

While multiple mechanisms likely contribute to the etiology and progression of neurodegeneration in AD, the pathogenic role of neuroinflammation is now well recognized and accepted. A sedentary lifestyle and unhealthy diet can accelerate the aging-associated chronic low-grade inflammatory process linked to neurodegeneration in AD. While a lack of pre-clinical models that can reliably mimic changes in both immune system and inflammatory machinery has complicated efforts to fully elucidate the underlying disease mechanism in a manner that would enable the manipulation of different pathways in order to delay the onset and/or progression of the disease, we describe a few that pivot on the inflammatory process (Figure 2). We discuss approaches including immunotherapeutic targeting immune checkpoint inhibitors, pharmaceuticals that selectively target the SASP microenvironment, and other detrimental aspects of neuroinflammation without affecting defense mechanisms against pathogens and tissue damage. Lifestyle changes that incorporate long-term EE regimen, CR/CR-mimetics and nutraceuticals, or a combination of these interventions may ameliorate inflammaging and its progression into AD. Remodeling the aging gut microbiota using probiotics or fecal transplants is likely to dampen the pro-inflammatory milleu and modulate the neurochemical and neuro-metabolic signaling pathways of the brain and protect against neuroinflammation. As there is a huge potential for therapies that modulate the neuroimmune response in AD, it is important to define the inflammatory stages to correlate to each phase of AD progression and clarify which processes are protective and which ones are detrimental, as well as identify suitable times, modes, and sites of intervention. This may facilitate a focused and functional therapeutic approach to alleviate age-related stress on the intracellular organelles, tissues, and systems and delay AD progression in aging.

## Abbreviations

AA	Arachidonic acid
Aβ	Amyloid beta
ABCA7	ATP-binding cassette subfamily A member 7
AD	Alzheimer’s disease
Ahr	Aryl hydrocarbon receptor
AMPK	AMP-activated kinase
APP	Amyloid precursor protein
BBB	Blood–brain barrier
BIN1	Bridging integrator 1
BMAA	α-Amino-β-methylaminopropionic acid
CCR2	C-C chemokine receptor type 2
CD	Cluster of differentiation
CD2AP	CD2 associated protein
CLU	Clusterin
CNS	Central nervous system
CR	Calorie restriction
CR1	Complement component (3b/4b) receptor 1
CRP	C-reactive protein
CSF	Cerebrospinal fluid
DAMPs	Damage associated molecular patterns
EE	Endurance exercise
EOAD	Early-onset Alzheimer’s disease
GFAP	Glial fibrillary acidic protein
GA	Glatiramer acetate
GWAS	Genome-wide association studies
HMGB1	High mobility group box 1
ICR	Inhibitory immune checkpoint receptor
IFNγ	Interferon gamma
IMiDs	Immunomodulatory imide drugs
LOAD	Late-onset Alzheimer’s disease
MDA	Malondialdehyde
MMP-9	Matrix metallopeptidase 9
Mo	Monocytes
MTOR	Mechanistic target of rapamycin
mtROS	Mitochondrial reactive oxygen species
MΦ	Macrophages
NFκB	Nuclear factor κB
NLRP3	Nod-like receptor 3
NOX	NADPH oxidase
OPC	Oligodendrocyte precursor cells
PD-1	Programmed cell death protein-1
PD-L1	Programmed cell death ligand-1
PICALM	Phosphatidylinositol binding clathrin assembly protein
PLD3	Phospholipase D3
PSEN1	Presenilin 1
PSEN2	Presenilin 2
ROI	Reactive oxygen intermediates
ROS	Reactive oxygen species
RONS	Reactive oxygen and nitrogen species
S6RP	S6 ribosomal protein
sAPP	Secreted amyloid precursor protein
SASP	Senescence-associated secretory phenotype
SCFA	Short-chain fatty acids
SORL1	Sortilin-related receptor-1
STAT3	Signal transducer and activators of transcription 3
T2DM	Type 2 diabetes mellitus
TGF-β	Transforming growth factor beta
TLR	Toll-like receptor
TNF-α	Tumor necrosis factor alpha
TNFR-I	Tumor necrosis factor receptor I
TNFR-II	Tumor necrosis factor receptor II
TRAIL	Tumor necrosis factor-related apoptosis inducing ligand
Treg	Regulatory T cells
TREM2	Triggering receptor expressed on myeloid cells 2
VCAM-I	Vascular cell adhesion molecule I
WES	Whole exome sequencing
WGS	Whole genome sequencing
XO	Xanthine oxidase

## References

[1] Pattabiraman G., Palasiewicz K., Galvin J.P., Ucker D.S. (2017). Aging-associated dysregulation of homeostatic immune response termination (and not initiation). Aging Cell.

[2] López-Otín C., Blasco M.A., Partridge L., Serrano M., Kroemer G. (2013). The Hallmarks of Aging. Cell.

[3] Spinelli R., Parrillo L., Longo M., Florese P., Desiderio A., Zatterale F., Miele C., Raciti G.A., Beguinot F. (2020). Molecular basis of ageing in chronic metabolic diseases. J. Endocrinol. Investig..

[4] Franceschi C., Bonafè M., Valensin S., Olivieri F., De Luca M., Ottaviani E., De Benedictis G. (2000). Inflamm-aging: An evolutionary perspective on immunosenescence. Ann. N. Y. Acad. Sci..

[5] Li T., Huang Y., Cai W., Chen X., Men X., Lu T., Wu A., Lu Z. (2020). Age-related cerebral small vessel disease and inflammaging. Cell Death Dis..

[6] Di Micco R., Krizhanovsky V., Baker D., d’Adda di Fagagna F. (2021). Cellular senescence in ageing: From mechanisms to therapeutic opportunities. Nat. Rev. Mol. Cell Biol..

[7] Fulop T., Larbi A., Dupuis G., Le Page A., Frost E.H., Cohen A.A., Witkowski J.M., Franceschi C. (2018). Immunosenescence and Inflamm-Aging As Two Sides of the Same Coin: Friends or Foes?. Front. Immunol..

[8] Franceschi C., Santoro A., Capri M. (2020). The complex relationship between Immunosenescence and Inflammaging: Special issue on the New Biomedical Perspectives. Semin. Immunopathol..

[9] Aiello A., Farzaneh F., Candore G., Caruso C., Davinelli S., Gambino C.M., Ligotti M.E., Zareian N., Accardi G. (2019). Immunosenescence and Its Hallmarks: How to Oppose Aging Strategically? A Review of Potential Options for Therapeutic Intervention. Front. Immunol..

[10] Barbé-Tuana F., Funchal G., Schmitz C.R.R., Maurmann R.M., Bauer M.E. (2020). The interplay between immunosenescence and age-related diseases. Semin. Immunopathol..

[11] Conte M., Martucci M., Chiariello A., Franceschi C., Salvioli S. (2020). Mitochondria, immunosenescence and inflammaging: A role for mitokines?. Semin. Immunopathol..

[12] Haas R.H. (2019). Mitochondrial Dysfunction in Aging and Diseases of Aging. Biology.

[13] Salminen A., Kaarniranta K., Kauppinen A. (2012). Inflammaging: Disturbed interplay between autophagy and inflammasomes. Aging.

[14] Barbosa M.C., Grosso R.A., Fader C.M. (2019). Hallmarks of Aging: An Autophagic Perspective. Front. Endocrinol..

[15] Picca A., Lezza A.M.S., Leeuwenburgh C., Pesce V., Calvani R., Landi F., Bernabei R., Marzetti E. (2017). Fueling Inflamm-Aging through Mitochondrial Dysfunction: Mechanisms and Molecular Targets. Int. J. Mol. Sci..

[16] Tran M., Reddy P.H. (2021). Defective Autophagy and Mitophagy in Aging and Alzheimer’s Disease. Front. Neurosci..

[17] Lopez-Castejon G. (2020). Control of the inflammasome by the ubiquitin system. FEBS J..

[18] Hegde A.N., Smith S.G., Duke L.M., Pourquoi A., Vaz S. (2019). Perturbations of Ubiquitin-Proteasome-Mediated Proteolysis in Aging and Alzheimer’s Disease. Front. Aging Neurosci..

[19] Ioannidou A., Goulielmaki E., Garinis G.A. (2016). DNA Damage: From Chronic Inflammation to Age-Related Deterioration. Front. Genet..

[20] da Silva P.F.L., Schumacher B. (2019). DNA damage responses in ageing. Open Biol..

[21] Chen G., Yung R. (2019). Meta-inflammaging at the crossroad of geroscience. Aging Med..

[22] Herradon G., Ramos-Alvarez M.P., Gramage E. (2019). Connecting Metainflammation and Neuroinflammation Through the PTN-MK-RPTPβ/ζ Axis: Relevance in Therapeutic Development. Front. Pharmacol..

[23] Vitale G., Salvioli S., Franceschi C. (2013). Oxidative stress and the ageing endocrine system. Nat. Rev. Endocrinol..

[24] Shintouo C.M., Mets T., Beckwee D., Bautmans I., Ghogomu S.M., Souopgui J., Leemans L., Meriki H.D., Njemini R. (2020). Is inflammageing influenced by the microbiota in the aged gut? A systematic review. Exp. Gerontol..

[25] Santoro A., Zhao J., Wu L., Carru C., Biagi E., Franceschi C. (2020). Microbiomes other than the gut: Inflammaging and age-related diseases. Semin. Immunopathol..

[26] Tang Y., Fung E., Xu A., Lan H.-Y. (2017). C-reactive protein and ageing. Clin. Exp. Pharmacol. Physiol..

[27] Rea I.M., Gibson D.S., McGilligan V., McNerlan S.E., Alexander H.D., Ross O.A. (2018). Age and Age-Related Diseases: Role of Inflammation Triggers and Cytokines. Front. Immunol..

[28] Marcos-Pérez D., Sánchez-Flores M., Proietti S., Bonassi S., Costa S., Teixeira J.P., Fernández-Tajes J., Pásaro E., Laffon B., Valdiglesias V. (2020). Association of inflammatory mediators with frailty status in older adults: Results from a systematic review and meta-analysis. GeroScience.

[29] Tchalla A.E., Wellenius G.A., Travison T.G., Gagnon M., Iloputaife I., Dantoine T., Sorond F.A., Lipsitz L.A. (2015). Circulating Vascular Cell Adhesion Molecule-1 Is Associated With Cerebral Blood Flow Dysregulation, Mobility Impairment, and Falls in Older Adults. Hypertension.

[30] Prochaska J.H., Frank B., Nagler M., Lamparter H., Weißer G., Schulz A., Eggebrecht L., Göbel S., Arnold N., Panova-Noeva M. (2017). Age-related diagnostic value of D-dimer testing and the role of inflammation in patients with suspected deep vein thrombosis. Sci. Rep..

[31] Lee S.-H., Lee J.-H., Lee H.-Y., Min A.K.-J. (2019). Sirtuin signaling in cellular senescence and aging. BMB Rep..

[32] Franceschi C., Campisi J. (2014). Chronic inflammation (inflammaging) and its potential contribution to age-associated diseases. J. Gerontol. A Biol. Sci. Med. Sci..

[33] Kennedy B.K., Berger S.L., Brunet A., Campisi J., Cuervo A.M., Epel E.S., Franceschi C., Lithgow G.J., Morimoto R.I., Pessin J.E. (2014). Geroscience: Linking Aging to Chronic Disease. Cell.

[34] Chen W.-W., Zhang X., Huang W.-J. (2016). Role of neuroinflammation in neurodegenerative diseases (Review). Mol. Med. Rep..

[35] Gross A.L., Walker K.A., Moghekar A.R., Pettigrew C., Soldan A., Albert M.S., Walston J.D. (2019). Plasma Markers of Inflammation Linked to Clinical Progression and Decline During Preclinical AD. Front. Aging Neurosci..

[36] Kuo C.-Y., Stachiv I., Nikolai T. (2020). Association of Late Life Depression, (Non-) Modifiable Risk and Protective Factors with Dementia and Alzheimer’s Disease: Literature Review on Current Evidences, Preventive Interventions and Possible Future Trends in Prevention and Treatment of Dementia. Int. J. Environ. Res. Public Health.

[37] Hou Y., Dan X., Babbar M., Wei Y., Hasselbalch S.G., Croteau D.L., Bohr V.A. (2019). Ageing as a risk factor for neurodegenerative disease. Nat. Rev. Neurol..

[38] Cummings J. (2021). New approaches to symptomatic treatments for Alzheimer’s disease. Mol. Neurodegener..

[39] Yiannopoulou K.G., Papageorgiou S.G. (2013). Current and future treatments for Alzheimer’s disease. Ther. Adv. Neurol. Disord..

[40] Deture M.A., Dickson D.W. (2019). The neuropathological diagnosis of Alzheimer’s disease. Mol. Neurodegener..

[41] Masters C.L., Bateman R., Blennow K., Rowe C.C., Sperling R.A., Cummings J.L. (2015). Alzheimer’s disease. Nat. Rev. Dis. Primers.

[42] Roda A.R., Montoliu-Gaya L., Serra-Mir G., Villegas S. (2020). Both Amyloid-β Peptide and Tau Protein Are Affected by an Anti-Amyloid-β Antibody Fragment in Elderly 3xTg-AD Mice. Int. J. Mol. Sci..

[43] Reitz C., Rogaeva E., Beecham G.W. (2020). Late-onset vs nonmendelian early-onset Alzheimer disease: A distinction without a difference?. Neurol. Genet..

[44] Reitz C., Mayeux R. (2014). Alzheimer disease: Epidemiology, diagnostic criteria, risk factors and biomarkers. Biochem. Pharmacol..

[45] Cruchaga C., Del-Aguila J.L., Saef B., Black K., Fernandez M.V., Budde J., Ibanez L., Deming Y., Kapoor M., Tosto G. (2018). Polygenic risk score of sporadic late-onset Alzheimer’s disease reveals a shared architecture with the familial and early-onset forms. Alzheimer’s Dement..

[46] Kelleher R.J., Shen J. (2017). Presenilin-1 mutations and Alzheimer’s disease. Proc. Natl. Acad. Sci. USA.

[47] A An S.S., Cai Y., Kim S. (2015). Mutations in presenilin 2 and its implications in Alzheimer’s disease and other dementia-associated disorders. Clin. Interv. Aging.

[48] Tambini M.D., A Norris K., D’Adamio L. (2020). Opposite changes in APP processing and human Aβ levels in rats carrying either a protective or a pathogenic APP mutation. eLife.

[49] Vidal C., Zhang L. (2021). An Analysis of the Neurological and Molecular Alterations Underlying the Pathogenesis of Alzheimer’s Disease. Cells.

[50] Tang M., Ryman D.C., McDade E., Jasielec M.S., Buckles V.D., Cairns N.J., Faga A.M., Goate A., Marcus D.S., Xiong C. (2016). Neurological manifestations of autosomal dominant familial Alzheimer’s disease: A comparison of the published literature with the dominantly inherited Alzheimer network observational study (DIAN-OBS). Lancet Neurol..

[51] Guerreiro R., Bras J. (2015). The age factor in Alzheimer’s disease. Genome Med..

[52] Corder E.H., Saunders A.M., Strittmatter W.J., Schmechel D.E., Gaskell P.C., Small G.W., Roses A.D., Haines J.L., Pericak-Vance M.A. (1993). Gene dose of apolipoprotein E type 4 allele and the risk of Alzheimer’s disease in late onset families. Science.

[53] Naj A.C., Schellenberg G.D. (2017). Alzheimer’s Disease Genetics Consortium (ADGC). Genomic variants, genes, and pathways of Alzheimer’s disease: An overview. Am. J. Med. Genet. B Neuropsychiatr. Genet..

[54] Prokopenko D., Morgan S.L., Mullin K., Hofmann O., Chapman B., Kirchner R., Amberkar S., Wohlers I., Lange C., Hide W. (2021). Whole-genome sequencing reveals new Alzheimer’s disease–associated rare variants in loci related to synaptic function and neuronal development. Alzheimer’s Dement..

[55] Karch C.M., Goate A.M. (2015). Alzheimer’s disease risk genes and mechanisms of disease pathogenesis. Biol. Psychiatry.

[56] Grozeva D., Saad S., Menzies G.E., Sims R. (2019). Benefits and Challenges of Rare Genetic Variation in Alzheimer’s Disease. Curr. Genet. Med. Rep..

[57] Lord J., Lu A.J., Cruchaga C. (2014). Identification of rare variants in Alzheimer’s disease. Front. Genet..

[58] Lambert J.C., Ibrahim-Verbaas C.A., Harold D., Naj A.C., Sims R., Bellenguez C., DeStafano A.L., Bis J.C., Beecham G.W., Grenier-Boley B. (2013). Meta-Analysis of 74,046 individuals identifies 11 new susceptibility loci for Alzheimer’s disease. Nat. Genet..

[59] Sims R., van der Lee S.J., Naj A.C., Bellenguez C., Badarinarayan N., Jakobsdottir J., Kunkle B.W., Boland A., Raybould R., Bis J.C. (2017). Rare coding variants in PLCG2, ABI3, and TREM2 implicate microglial-mediated innate immunity in Alzheimer’s disease. Nat. Genet..

[60] Efthymiou A.G., Goate A.M. (2017). Late onset Alzheimer’s disease genetics implicates microglial pathways in disease risk. Mol. Neurodegener..

[61] Gratuze M., Leyns C.E.G., Holtzman D.M. (2018). New insights into the role of TREM2 in Alzheimer’s disease. Mol. Neurodegener..

[62] Shi Y., Holtzman D.M. (2018). Interplay between innate immunity and Alzheimer disease: APOE and TREM2 in the spotlight. Nat. Rev. Immunol..

[63] Klein H.-U., McCabe C., Gjoneska E., Sullivan S.E., Kaskow B.J., Tang A., Smith R.V., Xu J., Pfenning A.R., Bernstein B.E. (2019). Epigenome-wide study uncovers large-scale changes in histone acetylation driven by tau pathology in aging and Alzheimer’s human brains. Nat. Neurosci..

[64] Lin Y.T., Seo J., Gao F., Feldman H.M., Wen H.L., Penney J., Cam H.P., Gjoneska E., Raja W.K., Cheng J. (2018). APOE4 causes widespread molecular and cellular alterations associated with Alzheimer’s disease phenotypes in human iPSC-derived brain cell types. Neuron.

[65] Gambhir I., Misra A., Chakrabarti S. (2018). New genetic players in late-onset Alzheimer’s disease: Findings of genome-wide association studies. Indian J. Med Res..

[66] Selkoe D.J., Hardy J. (2016). The amyloid hypothesis of Alzheimer’s disease at 25 years. EMBO Mol. Med..

[67] Guerreiro R., Wojtas A., Bras J., Carrasquillo M.M., Rogaeva E., Majounie E., Cruchaga C., Sassi C., Kauwe J.S., Younkin S.G. (2013). TREM2 Variants in Alzheimer’s Disease. N. Engl. J. Med..

[68] Li J.-T., Zhang Y. (2018). TREM2 regulates innate immunity in Alzheimer’s disease. J. Neuroinflamm..

[69] Zheng H., Cheng B., Li Y., Li X., Chen X., Zhang Y.-W. (2018). TREM2 in Alzheimer’s Disease: Microglial Survival and Energy Metabolism. Front. Aging Neurosci..

[70] Zhong L., Chen X.-F., Wang T., Wang Z., Liao C., Wang Z., Huang R., Wang D., Li X., Wu L. (2017). Soluble TREM2 induces inflammatory responses and enhances microglial survival. J. Exp. Med..

[71] Hickman S.E., El Khoury J. (2014). TREM2 and the neuroimmunology of Alzheimer’s disease. Biochem. Pharmacol..

[72] Gong C.-X., Liu F., Iqbal K. (2018). Multifactorial Hypothesis and Multi-Targets for Alzheimer’s Disease. J. Alzheimer’s Dis..

[73] De Roeck A., Van Broeckhoven C., Sleegers K. (2019). The role of ABCA7 in Alzheimer’s disease: Evidence from genomics, transcriptomics and methylomics. Acta Neuropathol..

[74] Iqbal K., Grundke-Iqbal I. (2010). Alzheimer’s disease, a multifactorial disorder seeking multitherapies. Alzheimer's Dement..

[75] Heppner F.L., Ransohoff R.M., Becher B. (2015). Immune attack: The role of inflammation in Alzheimer disease. Nat. Rev. Neurosci..

[76] Sala Frigerio C., Wolfs L., Fattorelli N., Thrupp N., Voytyuk I., Schmidt I., Mancuso R., Chen W.T., Woodbury M.E., Srivastava G. (2019). The Major Risk Factors for Alzheimer’s Disease: Age, Sex, and Genes Modulate the Microglia Response to Aβ Plaques. Cell Rep..

[77] Ising C., Venegas C., Zhang S., Scheiblich H., Schmidt S.V., Vieira-Saecker A., Schwartz S., Albasset S., McManus R.M., Tejera D. (2019). NLRP3 inflammasome activation drives tau pathology. Nature.

[78] Shen Z., Bao X., Wang R. (2018). Clinical PET Imaging of Microglial Activation: Implications for Microglial Therapeutics in Alzheimer’s Disease. Front. Aging Neurosci..

[79] Yao K., Zu H.B. (2020). Microglial polarization: Novel therapeutic mechanism against Alzheimer’s disease. Inflammopharmacology.

[80] Tondo G., Iaccarino L., Caminiti S.P., Presotto L., Santangelo R., Iannaccone S., Magnani G., Perani D. (2020). The combined effects of microglia activation and brain glucose hypometabolism in early-onset Alzheimer’s disease. Alzheimer’s Res. Ther..

[81] Wang W.-Y., Tan M.-S., Yu J.T., Tan L. (2015). Role of pro-inflammatory cytokines released from microglia in Alzheimer’s disease. Ann. Transl. Med..

[82] Stamouli E.C., Politis A.M. (2016). Pro-inflammatory cytokines in Alzheimer’s disease. Psychiatriki.

[83] Wang M.-M., Miao D., Cao X.-P., Tan L., Tan L. (2018). Innate immune activation in Alzheimer’s disease. Ann. Transl. Med..

[84] Fernández-Arjona M.D.M., Grondona J.M., Fernández-Llebrez P., López-Ávalos M.D. (2019). Microglial Morphometric Parameters Correlate With the Expression Level of IL-1β, and Allow Identifying Different Activated Morphotypes. Front. Cell Neurosci..

[85] Akiyama H., Barger S., Barnum S., Bradt B., Bauer J., Cole G.M., Cooper N.R., Eikelenboom P., Emmerling M., Fiebich B.L. (2000). Inflammation and Alzheimer’s disease. Neurobiol. Aging.

[86] Azizi G., Navabi S.S., Al-Shukaili A., Seyedzadeh M.H., Yazdani R., Mirshafiey A. (2015). The Role of Inflammatory Mediators in the Pathogenesis of Alzheimer’s Disease. Sultan Qaboos Univ. Med. J..

[87] Chen X.Q., Mobley W.C. (2019). Alzheimer Disease Pathogenesis: Insights From Molecular and Cellular Biology Studies of Oligomeric Aβ and Tau Species. Front. Neurosci..

[88] Heneka M.T., Carson M.J., El Khoury J., Landreth G.E., Brosseron F., Feinstein D.L., Jacobs A.H., Wyss-Coray T., Vitorica J., Ransohoff R.M. (2015). Neuroinflammation in Alzheimer’s disease. Lancet Neurol..

[89] Rios M.A.E., Etoral-Rios D., Efranco-Bocanegra D., Evilleda-Hernández J., Ecampos-Peña V. (2013). Inflammatory process in Alzheimer’s Disease. Front. Integr. Neurosci..

[90] Businaro R., Corsi M., Asprino R., Di Lorenzo C., Laskin D., Corbo R., Ricci S., Pinto A. (2018). Modulation of Inflammation as a Way of Delaying Alzheimer’s Disease Progression: The Diet’s Role. Curr. Alzheimer Res..

[91] Skaper S.D., Facci L., Zusso M., Giusti P. (2018). An Inflammation-Centric View of Neurological Disease: Beyond the Neuron. Front. Cell Neurosci..

[92] Duran-Aniotz C., Hetz C. (2016). Glucose Metabolism: A Sweet Relief of Alzheimer’s Disease. Curr. Biol..

[93] Tönnies E., Trushina E. (2017). Oxidative Stress, Synaptic Dysfunction, and Alzheimer’s Disease. J. Alzheimer's Dis..

[94] Thal D.R., Capetillo-Zarate E., Larionov S., Staufenbiel M., Zurbruegg S., Beckmann N. (2009). Capillary cerebral amyloid angiopathy is associated with vessel occlusion and cerebral blood flow disturbances. Neurobiol. Aging.

[95] Potter H., Granic A., Caneus J. (2015). Role of Trisomy 21 Mosaicism in Sporadic and Familial Alzheimer’s Disease. Curr. Alzheimer Res..

[96] Clare R., King V.G., Wirenfeldt M., Vinters H.V. (2010). Synapse loss in dementias. J. Neurosci. Res..

[97] Wang W., Zhao F., Ma X., Perry G., Zhu X. (2020). Mitochondria dysfunction in the pathogenesis of Alzheimer’s disease: Recent advances. Mol. Neurodegener..

[98] Norris G.T., Kipnis J. (2019). Immune cells and CNS physiology: Microglia and beyond. J. Exp. Med..

[99] Bennett M.L., Bennett F.C., Liddelow S.A., Ajami B., Zamanian J.L., Fernhoff N.B., Mulinyawe S.B., Bohlen C.J., Adil A., Tucker A. (2016). New tools for studying microglia in the mouse and human CNS. Proc. Natl. Acad. Sci. USA.

[100] Buttgereit A., Lelios I., Yu X., Vrohlings M., Krakoski N.R., Gautier E.L., Nishinakamura R., Becher B., Greter M. (2016). Sall1 is a transcriptional regulator defining microglia identity and function. Nat. Immunol..

[101] Gomez Perdiguero E., Klapproth K., Schulz C., Busch K., Azzoni E., Crozet L., Garner H., Trouillet C., de Bruijn M.F., Geissmann F. (2014). Tissue-resident macrophages originate from yolk-sac-derived erythro-myeloid progenitors. Nature.

[102] Gosselin D., Link V.M., Romanoski C.E., Fonseca G.J., Eichenfield D.Z., Spann N.J., Stender J.D., Chun H.B., Garner H., Geissmann F. (2014). Environment drives selection and function of enhancers controlling tissue-specific macrophage identities. Cell.

[103] Sheng J., Ruedl C., Karjalainen K. (2015). Most tissue-resident macrophages except microglia are derived from fetal hematopoietic stem cells. Immunity.

[104] Hoeffel G., Chen J., Lavin Y., Low D., Almeida F.F., See P., Beaudin A.E., Lum J., Low I., Forsberg E.C. (2015). C-Myb+ erythro-myeloid progenitor-derived fetal monocytes give rise to adult tissue-resident macrophages. Immunity.

[105] Ajami B., Bennett J.L., Krieger C., Tetzlaff W., Rossi F.M. (2007). Local self-renewal can sustain CNS microglia maintenance and function throughout adult life. Nat. Neurosci..

[106] Tay T.L., Hagemeyer N., Prinz M. (2016). The force awakens: Insights into the origin and formation of microglia. Curr. Opin. Neurobiol..

[107] Elmore M.R.P., Najafi A.R., Koike M.A., Dagher N.N., Spangenberg E.E., Rice R.A., Kitazawa M., Matusow B., Nguyen H., West B.L. (2014). Colony-stimulating factor 1 receptor signaling is necessary for microglia viability, unmasking a microglia progenitor cell in the adult brain. Neuron.

[108] Lenz K.M., Nelson L.H. (2018). Microglia and Beyond: Innate Immune Cells As Regulators of Brain Development and Behavioral Function. Front. Immunol..

[109] Mildner A., Schmidt H., Nitsche M., Merkler D., Hanisch U.-K., Mack M., Heikenwalder M., Brück W., Priller J., Prinz M. (2007). Microglia in the adult brain arise from Ly-6ChiCCR2+ monocytes only under defined host conditions. Nat. Neurosci..

[110] Wohleb E.S., Powell N.D., Godbout J.P., Sheridan J.F. (2013). Stress-induced recruitment of bone marrow-derived monocytes to the brain promotes anxiety-like behavior. J. Neurosci..

[111] Sochoka M., Diniz B.S., Leszek J. (2017). Inflammatory responses in the CNS: Fried or foe?. Mol. Neurobiol..

[112] Tejera D., Heneka M.T. (2016). Microglia in Alzheimer’s disease: The good, the bad and the ugly. Curr. Alzheimer Res..

[113] Wyss-Coray T., Rogers J. (2012). Inflammation in Alzheimer disease-a brief review of the basic science and clinical literature. Cold Spring Harbor Perspect. Med..

[114] Biber K., Neumann H., Inoue K., Boddeke H.W.G.M. (2007). Neuronal ‘On’ and ‘Off’ signals control microglia. Trends Neurosci..

[115] Baroja-Mazo A., Martín-Sánchez F., Gomez A.I., Martínez C.M., Amores-Iniesta J., Compan V., Barberà-Cremades M., Yagüe J., Ruiz-Ortiz E., Antón J. (2014). The NLRP3 inflammasome is released as a particulate danger signal that amplifies the inflammatory response. Nat. Immunol..

[116] Koenigsknecht-Talboo J., Landreth G.E. (2005). Microglial phagocytosis induced by fibrillar beta-amyloid and IgGs are differentially regulated by proinflammatory cytokines. J. Neurosci..

[117] Sondag C.M., Dhawan G., Combs C.K. (2009). Beta amyloid oligomers and fibrils stimulate differential activation of primary microglia. J. Neuroinflamm..

[118] Hommet C., Mondon K., Camus V., Ribeiro M.J., Beaufils E., Arlicot N., Corcia P., Paccalin M., Minier F., Gosselin T. (2014). Neuroinflammation and β Amyloid Deposition in Alzheimer’s Disease: In vivo Quantification with Molecular Imaging. Dement. Geriatr. Cogn. Disord..

[119] Nagele R.G., Wegiel J., Venkataraman V., Imaki H., Wang K.C., Wegiel J. (2004). Contribution of glial cells to the development of amyloid plaques in Alzheimer’s disease. Neurobiol. Aging.

[120] Serrano-Pozo A., Mielke M.L., Gómez-Isla T., Betensky R.A., Growdon J.H., Frosch M.P., Hyman B.T. (2020). Reactive glia not only associates with plaques but also parallels tangles in Alzheimer’s disease. Am. J. Pathol..

[121] Minter M.R., Taylor J.M., Crack P.J. (2016). The contribution of neuroinflammation to amyloid toxicity in Alzheimer’s disease. J. Neurochem..

[122] Bamberger M.E., Harris M.E., McDonald D.R., Husemann J., Landreth G.E. (2003). A cell surface receptor complex for fibrillar beta-amyloid mediates microglial activation. J. Neurosci..

[123] Ries M., Sastre M. (2016). Mechanisms of Aβ clearance and degradation by glial cells. Front. Aging Neurosci..

[124] Tahara K., Kim H.-D., Jin J.-J., Maxwell J.A., Li L., Fukuchi K. (2006). Role of toll-like receptor signalling in Abeta uptake and clearance. Brain J. Neurol..

[125] Wilkinson K., El Khoury J. (2012). Microglial scavenger receptors and their roles in the pathogenesis of Alzheimer’s disease. Int. J. Alzheimer's Dis..

[126] Hickman S.E., Allison E.K., El Khoury J. (2008). Microglial dysfunction and defective beta-amyloid clearance pathways in aging Alzheimer’s disease mice. J. Neurosci..

[127] Thériault P., ElAli A., Rivest S. (2015). The dynamics of monocytes and microglia in Alzheimer’s disease. Alzheimer's Res. Ther..

[128] Weiner H.L., Frenkel D. (2006). Immunology and immunotherapy of Alzheimer’s disease. Nat. Rev. Immunol..

[129] Frackowiak J., Wisniewski H.M., Wegiel J., Merz G.S., Iqbal K., Wang K.C. (1992). Ultrastructure of the microglia that phagocytose amyloid and the microglia that produce beta-amyloid fibrils. Acta Neuropathol..

[130] Krabbe G., Halle A., Matyash V., Rinnenthal J.L., Eom G.D., Bernhardt U., Miller K.R., Prokop S., Kettenmann H., Heppner F.L. (2013). Functional impairment of microglia coincides with beta-amyloid deposition in mice with Alzheimer-like pathology. PLoS ONE.

[131] Nagele R.G., D’Andrea M.R., Lee H., Venkataraman V., Wang H.Y. (2003). Astrocytes accumulate A beta 42 and give rise to astrocytic amyloid plaques in Alzheimer disease brains. Brain Res..

[132] Heneka M.T., Sastre M., Dumitrescu-Ozimek L., Hanke A., Dewachter I., Kuiperi C., O’Banion K., Klockgether T., Van Leuven F., Landreth G.E. (2005). Acute treatment with the PPARgamma agonist pioglitazone and ibuprofen reduces glial inflammation and Abeta1-42 levels in APPV717I transgenic mice. Brain.

[133] Meda L., Baron P., Scarlato G. (2001). Glial activation in Alzheimer’s disease: The role of Abeta and its associated proteins. Neurobiol. Aging.

[134] Morgan D., Gordon M.N., Tan J., Wilcock D., Rojiani A.M. (2005). Dynamic complexity of the microglial activation response in transgenic models of amyloid deposition: Implications for Alzheimer therapeutics. J. Neuropathol. Exp. Neurol..

[135] Tuppo E.E., Arias H.R. (2005). The role of inflammation in Alzheimer’s disease. Int. J. Biochem. Cell Biol..

[136] Tan Z.S., Seshadri S. (2010). Inflammation in the Alzheimer’s disease cascade: Culprit or innocent bystander?. Alzheimer's Res. Ther..

[137] Joshi P.G., Turola E., Ruiz A., Bergami A., Libera D.D., Benussi L., Giussani P., Magnani G., Comi G., Legname G. (2014). Microglia convert aggregated amyloid-β into neurotoxic forms through the shedding of microvesicles. Cell Death Differ..

[138] Hansen D.V., Hanson J.E., Sheng M. (2018). Microglia in Alzheimer’s disease. J. Cell Biol..

[139] Jiang C., Zou X., Zhu R., Shi Y., Wu Z., Zhao F., Chen L. (2018). The correlation between accumulation of amyloid beta with enhanced neuroinflammation and cognitive impairment after intraventricular hemorrhage. J. Neurosurg..

[140] Edison P., Archer H.A., Gerhard A., Hinz R., Pavese N., Turkheimer F.E., Hammers A., Tai Y.F., Fox N., Kennedy A. (2008). Microglia, amyloid, and cognition in Alzheimer’s disease: An [11C](R)PK11195-PET and [11C]PIB-PET study. Neurobiol. Dis..

[141] Okello A., Edison P., Archer H.A., Turkheimer F., Kennedy J., Bullock R., Walker Z., Fox N., Rossor M., Brooks D.J. (2009). Microglial activation and amyloid deposition in mild cognitive impairment: A PET study. Neurology.

[142] Hamelin L., Lagarde J., Dorothée G., Leroy C., Labit M., Comley R.A., De Souza L.C., Corne H., Dauphinot L., Bertoux M. (2016). Early and protective microglial activation in Alzheimer’s disease: A prospective study using18F-DPA-714 PET imaging. Brain.

[143] Fan Z., Okello A.A., Brooks D.J., Edison P. (2015). Longitudinal influence of microglial activation and amyloid on neuronal function in Alzheimer’s disease. Brain.

[144] Yokokura M., Mori N., Yagi S., Yoshikawa E., Kikuchi M., Yoshihara Y., Wakuda T., Sugihara G., Takebayashi K., Suda S. (2010). In vivo changes in microglial activation and amyloid deposits in brain regions with hypometabolism in Alzheimer’s disease. Eur. J. Nucl. Med. Mol. Imaging.

[145] Gosztyla M.L., Brothers H.M., Robinson S.R. (2018). Alzheimer’s Amyloid-β is an Antimicrobial Peptide: A Review of the Evidence. J. Alzheimer's Dis..

[146] Chung W.-S., Allen N.J., Eroglu C. (2015). Astrocytes Control Synapse Formation, Function, and Elimination. Cold Spring Harb. Perspect. Biol..

[147] Verkhratsky A., Nedergaard M. (2018). Physiology of Astroglia. Physiol. Rev..

[148] Preman P., Alfonso-Triguero M., Alberdi E., Verkhratsky A., Arranz A. (2021). Astrocytes in Alzheimer’s Disease: Pathological Significance and Molecular Pathways. Cells.

[149] Sheeler C., Rosa J.-G., Ferro A., McAdams B., Borgenheimer E., Cvetanovic M. (2020). Glia in Neurodegeneration: The Housekeeper, the Defender and the Perpetrator. Int. J. Mol. Sci..

[150] Gamage R., Wagnon I., Rossetti I., Childs R., Niedermayer G., Chesworth R., Gyengesi E. (2020). Cholinergic Modulation of Glial Function During Aging and Chronic Neuroinflammation. Front. Cell. Neurosci..

[151] Linnerbauer M., Wheeler M.A., Quintana F.J. (2020). Astrocyte Crosstalk in CNS Inflammation. Neuron.

[152] Giovannoni F., Quintana F.J. (2020). The role of astrocytes in CNS inflammation. Trends Immunol..

[153] Hol E.M., Pekny M. (2015). Glial fibrillary acidic protein (GFAP) and the astrocyte intermediate filament system in diseases of the central nervous system. Curr. Opin. Cell Biol..

[154] Yang Z., Wang K.K. (2015). Glial fibrillary acidic protein: From intermediate filament assembly and gliosis to neurobiomarker. Trends Neurosci..

[155] Beach T., McGeer E. (1988). Lamina-specific arrangement of astrocytic gliosis and senile plaques in Alzheimer’s disease visual cortex. Brain Res..

[156] Rodríguez J.J., Olabarria M., Chvatal A., Verkhratsky A., Rodr J.J. (2008). Astroglia in dementia and Alzheimer’s disease. Cell Death Differ..

[157] Verkhratsky A., Zorec R., Parpura V. (2017). Stratification of astrocytes in healthy and diseased brain. Brain Pathol..

[158] González-Reyes R.E., Nava-Mesa M.O., Vargas-Sánchez K., Ariza-Salamanca D., Mora-Muñoz L. (2017). Involvement of Astrocytes in Alzheimer’s Disease from a Neuroinflammatory and Oxidative Stress Perspective. Front. Mol. Neurosci..

[159] Colombo J., Quinn B., Puissant V. (2002). Disruption of astroglial interlaminar processes in Alzheimer’s disease. Brain Res. Bull..

[160] Yeh C.-Y., Vadhwana B., Verkhratsky A., Rodriguez J.J. (2011). Early Astrocytic Atrophy in the Entorhinal Cortex of a Triple Transgenic Animal Model of Alzheimer’s Disease. ASN Neuro.

[161] Beauquis J., Vinuesa A., Pomilio C., Pavía P., Galván V., Saravia F. (2014). Neuronal and Glial Alterations, Increased Anxiety, and Cognitive Impairment before Hippocampal Amyloid Deposition in PDAPP Mice, Model of Alzheimer’s Disease. Hippocampus.

[162] Diniz L.P., Tortelli V., Matias I., Morgado J., Araujo A.P.B., Melo H.M., Da Silva G.S.S., Alves-Leon S.V., De Souza J.M., Ferreira S.T. (2017). Astrocyte Transforming Growth Factor Beta 1 Protects Synapses against Aβ Oligomers in Alzheimer’s Disease Model. J. Neurosci..

[163] Iram T., Trudler D., Kain D., Kanner S., Galron R., Vassar R., Barzilai A., Blinder P., Fishelson Z., Frenkel D. (2016). Astrocytes from old Alzheimer’s disease mice are impaired in Aβ uptake and in neuroprotection. Neurobiol. Dis..

[164] Polis B., Srikanth K.D., Elliott E., Gil-Henn H., Samson A.O. (2018). L-Norvaline Reverses Cognitive Decline and Synaptic Loss in a Murine Model of Alzheimer’s Disease. Neurotherapeutics.

[165] Jones V.C., Atkinson-Dell R., Verkhratsky A., Mohamet L. (2017). Aberrant iPSC-Derived Human Astrocytes in Alzheimer’s Disease. Cell Death Dis..

[166] Kraft A.W., Hu X., Yoon H., Yan P., Xiao Q., Wang Y., Gil S.C., Brown J., Wilhelmsson U., Restivo J.L. (2013). Attenuating astrocyte activation accelerates plaque pathogenesis in APP/PS1 mice. FASEB J..

[167] Kobayashi E., Nakano M., Kubota K., Himuro N., Mizoguchi S., Chikenji T., Otani M., Mizue Y., Nagaishi K., Fujimiya M. (2018). Activated forms of astrocytes with higher GLT-1 expression are associated with cognitive normal subjects with Alzheimer pathology in human brain. Sci. Rep..

[168] Masliah E., Alford M., DeTeresa R., Mallory M., Hansen L. (1996). Deficient glutamate transport is associated with neurodegeneration in Alzheimer’s disease. Ann. Neurol..

[169] Scimemi A., Meabon J.S., Woltjer R.L., Sullivan J.M., Diamond J.S., Cook D.G. (2013). Amyloid-β1-42 slows clearance of synaptically released glutamate by mislocalizing astrocytic GLT-1. J. Neurosci..

[170] Vincent A.J., Gasperini R., Foa L., Small D.H. (2010). Astrocytes in Alzheimer’s Disease: Emerging Roles in Calcium Dysregulation and Synaptic Plasticity. J. Alzheimer’s Dis..

[171] Ettle B., Schlachetzki J.C.M., Winkler J. (2016). Oligodendroglia and myelin in neurodegenerative diseases: More than just bystanders?. Mol. Neurobiol..

[172] Mathys H., Davila-Velderrain J., Peng Z., Gao F., Mohammadi S., Young J.Z., Menon M., He L., Abdurrob F., Jiang X. (2019). Single-cell transcriptomic analysis of Alzheimer’s disease. Nature.

[173] Bartzokis G. (2004). Age-related myelin breakdown: A developmental model of cognitive decline and Alzheimer’s disease. Neurobiol. Aging.

[174] Bartzokis G., Lu P.H., Mintz J. (2004). Quantifying age-related myelin breakdown with MRI: Novel therapeutic targets for preventing cognitive decline and Alzheimer’s disease. J. Alzheimer's Dis..

[175] Mitew S., Kirkcaldie M.T.K., Halliday G.M., Shepherd C.E., Vickers J.C., Dickson T.C. (2010). Focal demyelination in Alzheimer’s disease and transgenic mouse models. Acta Neuropathol..

[176] Desai M.K., Mastrangelo M.A., Ryan D.A., Sudol K.L., Narrow W.C., Bowers W.J. (2010). Early Oligodendrocyte/Myelin Pathology in Alzheimer’s Disease Mice Constitutes a Novel Therapeutic Target. Am. J. Pathol..

[177] Goldmann T., Wieghofer P., Jordão M.J.C., Prutek F., Hagemeyer N., Frenzel K., Amann L., Staszewski O., Kierdorf K., Krueger M. (2016). Origin, fate and dynamics of macrophages at central nervous system interfaces. Nat. Immunol..

[178] Chinnery H.R., Ruitenberg M.J., McMenamin P.G. (2010). Novel characterization of monocyte-derived cell populations in the meninges and choroid plexus and their rates of replenishment in bone marrow chimeric mice. J. Neuropathol. Exp. Neurol..

[179] Prinz M., Priller J. (2014). Microglia and brain macrophages in the molecular age: From origin to neuropsychiatric disease. Nat. Rev. Neurosci..

[180] Mohamed A., Posse de Chaves E. (2011). Aβ internalization by neurons and glia. Int. J. Alzheimer's Dis..

[181] Simard A.R., Soulet D., Gowing G., Julien J.-P., Rivest S. (2006). Bone Marrow-Derived Microglia Play a Critical Role in Restricting Senile Plaque Formation in Alzheimer’s Disease. Neuron.

[182] Thanopoulou K., Fragkouli A., Stylianopoulou F., Georgopoulos S. (2010). Scavenger receptor class B type I (SR-BI) regulates perivascular macrophages and modifies amyloid pathology in an Alzheimer mouse model. Proc. Natl. Acad. Sci. USA.

[183] Town T., Laouar Y., Pittenger C., Mori T., Szekely C.A., Tan J., Duman R.S., Flavell R.A. (2008). Blocking TGF-beta-Smad2/3 innate immune signaling mitigates Alzheimer-like pathology. Nat. Med..

[184] El Khoury J., Toft M., Hickman S.E., Means T.K., Terada K., Geula C., Luster A.D. (2007). Ccr2 deficiency impairs microglial accumulation and accelerates progression of Alzheimer-like disease. Nat. Med..

[185] Anding A.L., Baehrecke E.H. (2017). Cleaning House: Selective Autophagy of Organelles. Dev. Cell.

[186] Li W., He P., Huang Y., Li Y.-F., Lu J., Li M., Kurihara H., Luo Z., Meng T., Onishi M. (2021). Selective autophagy of intracellular organelles: Recent research advances. Theranostics.

[187] Wang R., Wang G. (2019). Autophagy in Mitochondrial Quality Control. Adv. Exp. Med. Biol..

[188] Harper J.W., Ordureau A., Heo J.-M. (2018). Building and decoding ubiquitin chains for mitophagy. Nat. Rev. Mol. Cell Biol..

[189] Green D.R., Galluzzi L., Kroemer G. (2011). Mitochondria and the Autophagy-Inflammation-Cell Death Axis in Organismal Aging. Science.

[190] Green D.R., Levine B. (2014). To be or not to be? How selective autophagy and cell death govern cell fate. Cell. Mol. Life Sci..

[191] Desai S., Juncker M., Kim C. (2018). Regulation of mitophagy by the ubiquitin pathway in neurodegenerative diseases. Exp. Biol. Med..

[192] Madruga E., Maestro I., Martínez A. (2021). Mitophagy Modulation, a New Player in the Race against ALS. Int. J. Mol. Sci..

[193] Nakahira K., Haspel J.A., Rathinam V.A., Lee S.-J., Dolinay T., Lam H.C., Englert J.A., Rabinovitch M., Cernadas M., Kim H.P. (2011). Autophagy proteins regulate innate immune responses by inhibiting the release of mitochondrial DNA mediated by the NALP3 inflammasome. Nat. Immunol..

[194] Shimada K., Crother T.R., Karlin J., Dagvadorj J., Chiba N., Chen S., Ramanujan V.K., Wolf A.J., Vergnes L., Ojcius D.M. (2012). Oxidized Mitochondrial DNA Activates the NLRP3 Inflammasome during Apoptosis. Immunity.

[195] Dall’Olio F., Vanhooren V., Chen C.C., Slagboom P.E., Wuhrer M., Franceschi C. (2013). N-glycomic biomarkers of biological aging and longevity: A link with inflammaging. Ageing Res. Rev..

[196] Feldman N., Rotter-Maskowitz A., Okun E. (2015). DAMPs as mediators of sterile inflammation in aging-related pathologies. Ageing Res. Rev..

[197] Fang C., Wei X., Wei Y. (2016). Mitochondrial DNA in the regulation of innate immune responses. Protein Cell.

[198] Lamkanfi M., Dixit V.M. (2014). Mechanisms and functions of inflammasomes. Cell.

[199] Vanaja S.K., Rathinam V.A., Fitzgerald K.A. (2015). Mechanisms of inflammasome activation: Recent advances and novel insights. Trends Cell Biol..

[200] Mamik M.K., Power C. (2017). Inflammasomes in neurological diseases: Emerging pathogenic and therapeutic concepts. Brain.

[201] Swanson K.V., Deng M., Ting J.P.-Y. (2019). The NLRP3 inflammasome: Molecular activation and regulation to therapeutics. Nat. Rev. Immunol..

[202] Downs K.P., Nguyen H., Dorfleutner A., Stehlik C. (2020). An overview of the non-canonical inflammasome. Mol. Asp. Med..

[203] Horng T. (2014). Calcium signaling and mitochondrial destabilization in the triggering of the NLRP3 inflammasome. Trends Immunol..

[204] Harris J., Hartman M., Roche C., Zeng S.G., O’Shea A., Sharp F.A., Lambe E.M., Creagh E.M., Golenbock D.T., Tschopp J. (2011). Autophagy controls IL-1{beta} secretion by targeting pro-IL-1{beta} for degradation. J. Biol. Chem..

[205] Lee C., Kim K.H., Cohen P. (2016). MOTS-c: A novel mitochondrial-derived peptide regulating muscle and fat metabolism. Free. Radic. Biol. Med..

[206] Zhai D., Ye Z., Jiang Y., Xu C., Ruan B., Yang Y., Lei X., Xiang A., Lu H., Zhu Z. (2017). MOTS-c peptide increases survival and decreases bacterial load in mice infected with MRSA. Mol. Immunol..

[207] Oh Y.K., Bachar A.R., Zacharias D.G., Kim S.G., Wan J., Cobb L.J., Lerman L.O., Cohen P., Lerman A. (2011). Humanin preserves endothelial function and prevents atherosclerotic plaque progression in hypercholesterolemic ApoE deficient mice. Atherosclerosis.

[208] Sreekumar P.G., Ishikawa K., Spee C., Mehta H.H., Wan J., Yen K., Cohen P., Kannan R., Hinton D.R. (2016). The Mitochondrial-Derived Peptide Humanin Protects RPE Cells From Oxidative Stress, Senescence, and Mitochondrial Dysfunction. Investig. Opthalmology Vis. Sci..

[209] Muzumdar R.H., Huffman D.M., Atzmon G., Buettner C., Cobb L.J., Fishman S., Budagov T., Cui L., Einstein F.H., Poduval A. (2009). Humanin: A Novel Central Regulator of Peripheral Insulin Action. PLoS ONE.

[210] Halle A., Hornung V., Petzold G.C., Stewart C.R., Monks B.G., Reinheckel T., Fitzgerald K.A., Latz E., Moore K.J., Golenbock D.T. (2008). The NALP3 inflammasome is involved in the innate immune response to amyloid-beta. Nat. Immunol..

[211] Heneka M.T., Golenbock D.T., Latz E. (2015). Innate immunity in Alzheimer’s disease. Nat. Immunol..

[212] Kim M.-J., Yoon J.-H., Ryu J.-H. (2016). Mitophagy: A balance regulator of NLRP3 inflammasome activation. BMB Rep..

[213] Joshi A.U., Minhas P.S., Liddelow S.A., Haileselassie B., Andreasson K.I., Dorn G.W., Mochly-Rosen D. (2019). Fragmented mitochondria released from microglia trigger A1 astrocytic response and propagate inflammatory neurodegeneration. Nat. Neurosci..

[214] Fairley L.H., Wong J.H., Barron A.M. (2021). Mitochondrial Regulation of Microglial Immunometabolism in Alzheimer’s Disease. Front. Immunol..

[215] Koellhoffer E.C., McCullough L.D., Ritzel R.M. (2017). Old Maids: Aging and Its Impact on Microglia Function. Int. J. Mol. Sci..

[216] Agrawal I., Jha S. (2020). Mitochondrial Dysfunction and Alzheimer’s Disease: Role of Microglia. Front. Aging Neurosci..

[217] Baik S.H., Kang S., Lee W., Choi H., Chung S., Kim J.-I., Mook-Jung I. (2019). A Breakdown in Metabolic Reprogramming Causes Microglia Dysfunction in Alzheimer’s Disease. Cell Metab..

[218] Afridi R., Kim J.-H., Rahman H., Suk K. (2020). Metabolic Regulation of Glial Phenotypes: Implications in Neuron–Glia Interactions and Neurological Disorders. Front. Cell. Neurosci..

[219] Lauro C., Limatola C. (2020). Metabolic Reprograming of Microglia in the Regulation of the Innate Inflammatory Response. Front. Immunol..

[220] Afridi R., Lee W.H., Suk K. (2020). Microglia Gone Awry: Linking Immunometabolism to Neurodegeneration. Front. Cell Neurosci..

[221] Pan R.Y., Ma J., Kong X.X., Wang X.F., Li S.S., Qi X.L., Yan Y.H., Cheng J., Liu Q., Jin W. (2019). Sodium rutin ameliorates Alzheimer’s disease-like pathology by enhancing microglial amyloid-β clearance. Sci. Adv..

[222] Bonda D.J., Wang X., Lee H.-G., Smith M.A., Perry G., Zhu X. (2014). Neuronal failure in Alzheimer’s disease: A view through the oxidative stress looking-glass. Neurosci. Bull..

[223] Dorey E., Chang N., Liu Q.Y., Yang Z., Zhang W. (2014). Apolipoprotein E, amyloid-beta, and neuroinflammation in Alzheimer’s disease. Neurosci. Bull..

[224] Giasson B.I., Duda J.E., Murray I.V.J., Chen Q., Souza J.M., Hurtig H.I., Ischiropoulos H., Trojanowski J.Q., Lee V.M.Y. (2000). Oxidative damage linked to neurodegeneration by selective α-synuclein nitration in synucleinopathy lesions. Science.

[225] Fischer R., Maier O. (2015). Interrelation of oxidative stress and inflammation in neurodegenerative disease: Role of TNF. Oxid. Med. Cell. Longev..

[226] Cheeseman K.H., Slater T.F. (1993). An Introduction to Free-Radical Biochemistry. Br. Med. Bull..

[227] Marosi K., Bori Z., Hart N., Sárga L., Koltai E., Radák Z., Nyakas C. (2012). Long-term exercise treatment reduces oxidative stress in the hippocampus of aging rats. Neurosci..

[228] Liguori I., Russo G., Curcio F., Bulli G., Aran L., Della-Morte D., Gargiulo G., Testa G., Cacciatore F., Bonaduce D. (2018). Oxidative stress, aging, and diseases. Clin. Interv. Aging.

[229] Baeeri M., Bahadar H., Rahimifard M., Navaei-Nigjeh M., Khorasani R., Rezvanfar M.A., Gholami M., Abdollahi M. (2019). Alpha-Lipoic acid prevents senescence, cell cycle arrest, and inflammatory cues in fibroblasts by inhibiting oxidative stress. Pharm. Res..

[230] Chausse B., Lewen A., Poschet G., Kann O. (2020). Selective inhibition of mitochondrial respiratory complexes controls the transition of microglia into a neurotoxic phenotype in situ. Brain, Behav. Immun..

[231] Calvani R., Picca A., Marini F., Biancolillo A., Gervasoni J., Persichilli S., Primiano A., Coelho-Junior H.J., Bossola M., Urbani A. (2018). A Distinct Pattern of Circulating Amino Acids Characterizes Older Persons with Physical Frailty and Sarcopenia: Results from the BIOSPHERE Study. Nutrients.

[232] Disabato D.J., Quan N., Godbout J.P. (2016). Neuroinflammation: The devil is in the details. J. Neurochem..

[233] Baierle M., Nascimento S.N., Moro A.M., Brucker N., Freitas F., Gauer B., Durgante J., Bordignon S., Zibetti M., Trentini C.M. (2015). Relationship between Inflammation and Oxidative Stress and Cognitive Decline in the Institutionalized Elderly. Oxidative Med. Cell. Longev..

[234] Zuo L., Christofi F.L., Wright V.P., Bao S., Clanton T.L. (2004). Lipoxygenase-dependent superoxide release in skeletal muscle. J. Appl. Physiol..

[235] De la Fuente M., Miquel J. (2009). An update of the oxidation-inflammation theory of aging: The involvement of the immune system in oxi-inflamm-aging. Curr. Pharm. Des..

[236] Daulatzai M.A. (2017). Cerebral hypoperfusion and glucose hypometabolism: Key pathophysiological modulators promote neurodegeneration, cognitive impairment, and Alzheimer’s disease. J. Neurosci. Res..

[237] Campisi J., D’Adda Di Fagagna F. (2007). Cellular senescence: When bad things happen to good cells. Nat. Rev. Mol. Cell. Biol..

[238] Sharpless N.E., Sherr C.J. (2017). Forging a signature of in vivo senescence. Nat. Rev. Cancer.

[239] Cuollo L., Antonangeli F., Santoni A., Soriani A. (2020). The Senescence-Associated Secretory Phenotype (SASP) in the Challenging Future of Cancer Therapy and Age-Related Diseases. Biology.

[240] Acosta J.C., O’Loghlen A., Banito A., Guijarro M.V., Augert A., Raguz S., Fumagalli M., Da Costa M., Brown C., Popov N. (2008). Chemokine Signaling via the CXCR2 Receptor Reinforces Senescence. Cell.

[241] Coppé J.-P., Desprez P.-Y., Krtolica A., Campisi J. (2010). The Senescence-Associated Secretory Phenotype: The Dark Side of Tumor Suppression. Annu. Rev. Pathol. Mech. Dis..

[242] Neves J., Demaria M., Campisi J., Jasper H. (2015). Of Flies, Mice, and Men: Evolutionarily Conserved Tissue Damage Responses and Aging. Dev. Cell.

[243] Borodkina A.V., Deryabin P.I., Giukova A.A., Nikolsky N.N. (2018). ‘Social life’ of senescent sells: What is SASP and why study it?. Acta Naturae.

[244] Moreno-Blas D., Gorostieta-Salas E., Pommer-Alba A., Muciño-Hernández G., Gerónimo-Olvera C., Maciel-Barón L.A., Konigsberg M., Massieu L., Castro-Obregón S. (2019). Cortical neurons develop a senescence-like phenotype promoted by dysfunctional autophagy. Aging.

[245] Acklin S., Zhang M., Du W., Zhao X., Plotkin M., Chang J., Campisi J., Zhou D., Xia F. (2020). Depletion of senescent-like neuronal cells alleviates cisplatin-induced peripheral neuropathy in mice. Sci. Rep..

[246] Schmeer C., Kretz A., Wengerodt D., Stojiljkovic M., Witte O.W. (2019). Dissecting Aging and Senescence—Current Concepts and Open Lessons. Cells.

[247] McHugh D., Gil J. (2018). Senescence and aging: Causes, consequences, and therapeutic avenues. J. Cell Biol..

[248] Wengerodt D., Schmeer C., Witte O.W., Kretz A. (2019). Amitosenescence and Pseudomitosenescence: Putative New Players in the Aging Process. Cells.

[249] Bussian T.J., Aziz A., Meyer C.F., Swenson B.L., Van Deursen J.M., Baker D.J. (2018). Clearance of senescent glial cells prevents tau-dependent pathology and cognitive decline. Nat. Cell Biol..

[250] Musi N., Valentine J.M., Sickora K.R., Baeuerle E., Thompson C.S., Shen Q., Orr M.E. (2018). Tau protein aggregation is associated with cellular senescence in the brain. Aging Cell.

[251] Walton C.C., Andersen J.K. (2019). Unknown fates of (brain) oxidation or UFO: Close encounters with neuronal senescence. Free Radic. Biol. Med..

[252] Zhang P., Kishimoto Y., Grammatikakis I., Gottimukkala K., Cutler R.G., Zhang S., Abdelmohsen K., Bohr V.A., Sen J.M., Gorospe M. (2019). Senolytic therapy alleviates Aβ-associated oligodendrocyte progenitor cell senescence and cognitive deficits in an Alzheimer’s disease model. Nat. Neurosci..

[253] Basisty N., Kale A., Jeon O.H., Kuehnemann C., Payne T., Rao C., Holtz A., Shah S., Sharma V., Ferrucci L. (2020). A proteomic atlas of senescence-associated secretomes for aging biomarker development. PLoS Biol..

[254] Hernandez-Segura A., de Jong T.V., Melov S., Guryev V., Campisi J., Demaria M. (2017). Unmasking transcriptional heterogeneity in senescent cells. Curr. Biol..

[255] Fafián-Labora J.A., O’Loghlen A. (2020). Classical and Nonclassical Intercellular Communication in Senescence and Ageing. Trends Cell Biol..

[256] Herranz N., Gil J. (2018). Mechanisms and functions of cellular senescence. J. Clin. Investig..

[257] Song P., An J., Zou M.-H. (2020). Immune Clearance of Senescent Cells to Combat Ageing and Chronic Diseases. Cells.

[258] Prata L.G.L., Ovsyannikova I.G., Tchkonia T., Kirkland J.L. (2018). Senescent cell clearance by the immune system: Emerging therapeutic opportunities. Semin. Immunol..

[259] Ferrucci L., Fabbri E. (2018). Inflammageing: Chronic inflammation in ageing, cardiovascular disease, and frailty. Nat. Rev. Cardiol..

[260] Demirci D., Dayanc B., Mazi F., Senturk S. (2021). The Jekyll and Hyde of Cellular Senescence in Cancer. Cells.

[261] Walton C.C., Begelman D., Nguyen W., Andersen J.K. (2020). Senescence as an Amyloid Cascade: The Amyloid Senescence Hypothesis. Front. Cell. Neurosci..

[262] Carreno G., Guiho R., Martinez-Barbera J.P. (2021). Cell senescence in neuropathology: A focus on neurodegeneration and tumours. Neuropathol. Appl. Neurobiol..

[263] Bhat R., Crowe E.P., Bitto A., Moh M., Katsetos C.D., Garcia F.U., Johnson F.B., Trojanowski J.Q., Sell C., Torres C. (2012). Astrocyte senescence as a component of Alzheimer’s disease. PLoS ONE.

[264] Pertusa M., García-Matas S., Rodríguez-Farré E., Sanfeliu C., Cristòfol R. (2007). Astrocytes aged in vitro show a decreased neuroprotective capacity. J. Neurochem..

[265] Mansour H., Chamberlain C.G., Weible M.W.I., Hughes S., Chu Y., Chan-Ling T. (2008). Aging-related changes in astrocytes in the rat retina: Imbalance between cell proliferation and cell death reduces astrocyte availability. Aging Cell.

[266] Lee J.-H., Yu W.H., Kumar A., Lee S., Mohan P.S., Peterhoff C.M., Wolfe D.M., Martinez-Vicente M., Massey A.C., Sovak G. (2010). Lysosomal Proteolysis and Autophagy Require Presenilin 1 and Are Disrupted by Alzheimer-Related PS1 Mutations. Cell.

[267] Evans R.J., Wyllie F.S., Wynford-Thomas D., Kipling D., Jones C.J. (2003). A P53-dependent, telomere-independent proliferative life span barrier in human astrocytes consistent with the molecular genetics of glioma development. Cancer Res..

[268] Bitto A., Sell C., Crowe E., Lorenzini A., Malaguti M., Hrelia S., Torres C. (2010). Stress-induced senescence in human and rodent astrocytes. Exp. Cell Res..

[269] Oddo S.C.A., Shepherd J.D., Murphy M.P., Golde T.E., Kayed R., Metherate R., Mattson M.P., Akbari Y., LaFerla F.M. (2003). Triple-transgenic model of Alzheimer’s disease with plaques and tangles: Intracellular Abeta and synaptic dysfunction. Neuron.

[270] Vanzulli I., Papanikolaou M., De-La-Rocha I.C., Pieropan F., Rivera A.D., Gomez-Nicola D., Verkhratsky A., Rodríguez J.J., Butt A.M. (2020). Disruption of oligodendrocyte progenitor cells is an early sign of pathology in the triple transgenic mouse model of Alzheimer’s disease. Neurobiol. Aging.

[271] Luterman J.D., Haroutunian V., Yemul S., Ho L., Purohit D., Aisen P.S., Mohs R., Pasinetti G.M. (2000). Cytokine gene expression as a function of the clinical progression of Alzheimer disease dementia. Arch Neurol..

[272] Gruol D.L. (2015). IL-6 regulation of synaptic function in the CNS. Neuropharmacology.

[273] Iannello A., Thompson T.W., Ardolino M., Lowe S.W., Raulet D.H. (2013). p53-dependent chemokine production by senescent tumor cells supports NKG2D-dependent tumor elimination by natural killer cells. J. Exp. Med..

[274] Sagiv A., Burton D.G.A., Moshayev Z., Vadai E., Wensveen F., Ben-Dor S., Golani O., Polic B., Krizhanovsky V. (2016). NKG2D ligands mediate immunosurveillance of senescent cells. Aging.

[275] Pereira B.I., Devine O.P., Vukmanovic-Stejic M., Chambers E.S., Subramanian P., Patel N., Virasami A., Sebire N.J., Kinsler V., Valdovinos A. (2019). Senescent cells evade immune clearance via HLA-E-mediated NK and CD8+ T cell inhibition. Nat. Commun..

[276] Muñoz-Espín D., Cañamero M., Maraver A., Gómez-López G., Contreras J., Murillo-Cuesta S., Rodríguez-Baeza A., Varela-Nieto I., Ruberte J., Collado M. (2013). XProgrammed cell senescence during mammalian embryonic development. Cell.

[277] Kang T.-W., Yevsa T., Woller N., Hoenicke L., Wuestefeld T., Dauch D., Hohmeyer A., Gereke M., Rudalska R., Potapova A. (2011). Senescence surveillance of pre-malignant hepatocytes limits liver cancer development. Nature.

[278] Krizhanovsky V., Yon M., Dickins R.A., Hearn S., Simon J., Miething C., Yee H., Zender L., Lowe S.W. (2008). Senescence of Activated Stellate Cells Limits Liver Fibrosis. Cell.

[279] Galea I., Bechmann I., Perry V.H. (2007). What is immune privilege (not)?. Trends Immunol..

[280] Korin B., Ben-Shaanan T.L., Schiller M., Dubovik T., Azulay-Debby H., Boshnak N.T., Koren T., Rolls A. (2017). High-dimensional, single-cell characterization of the brain’s immune compartment. Nat. Neurosci..

[281] Benakis C., Llovera G., Liesz A. (2018). The meningeal and choroidal infiltration routes for leukocytes in stroke. Ther. Adv. Neurol. Disord..

[282] Ardura-Fabregat A., Boddeke E.W.G.M., Boza-Serrano A., Brioschi S., Castro-Gomez S., Ceyzériat K., Dansokho C., Dierkes T., Gelders G., Heneka M.T. (2017). Targeting Neuroinflammation to Treat Alzheimer’s Disease. CNS Drugs.

[283] Gorlé N., Van Cauwenberghe C., Libert C., Vandenbroucke R.E. (2016). The effect of aging on brain barriers and the consequences for Alzheimer’s disease development. Mamm. Genome.

[284] Sweeney M.D., Sagare A.P., Zlokovic B.V. (2018). Blood–brain barrier breakdown in Alzheimer disease and other neurodegenerative disorders. Nat. Rev. Neurol..

[285] Nation D.A., Sweeney M.D., Montagne A., Sagare A.P., D’Orazio L.M., Pachicano M., Sepehrband F., Nelson A.R., Buennagel D.P., Harrington M.G. (2019). Blood–brain barrier breakdown is an early biomarker of human cognitive dysfunction. Nat. Med..

[286] van de Haar H.J., Burgmans S., Jansen J.F., van Osch M.J., van Buchem M.A., Muller M., Hofman P.A., Verhey F.R., Backes W.H. (2016). Blood-Brain Barrier Leakage in Patients with Early Alzheimer Disease. Radiology.

[287] McManus R.M., Mills K.H., Lynch M.A. (2015). T cells—protective or pathogenic in Alzheimer’s disease?. J. Neuroimmune Pharmacol..

[288] Yu J.T., Xu W., Tan C.C., Andrieu S., Suckling J., Evangelou E., Pan A., Zhang C., Jia J., Feng L. (2020). Evidence-based prevention of Alzheimer’s disease: Systematic review and meta-analysis of 243 observational prospective studies and 153 randomised controlled trials. J. Neurol. Neurosurg. Psychiatry.

[289] Kivipelto M., Mangialasche F., Ngandu T. (2018). Lifestyle interventions to prevent cognitive impairment, dementia and Alzheimer disease. Nat. Rev. Neurol..

[290] Nguyen T.T., Ta Q.T.H., Nguyen T.K.O., Nguyen T.T.D., Giau V.V. (2020). Type 3 Diabetes and Its Role Implications in Alzheimer’s Disease. Int. J. Mol. Sci..

[291] Kandimalla R., Thirumala V., Reddy P.H. (2017). Is Alzheimer’s disease a Type 3 Diabetes? A critical appraisal. Biochim. Biophys. Acta (BBA) Mol. Basis Dis..

[292] Sun S., Ji Y., Kersten S., Qi L. (2012). Mechanisms of Inflammatory Responses in Obese Adipose Tissue. Annu. Rev. Nutr..

[293] Mraz M., Haluzik M. (2014). The role of adipose tissue immune cells in obesity and low-grade inflammation. J. Endocrinol..

[294] Schenk S., Saberi M., Olefsky J.M. (2008). Insulin sensitivity: Modulation by nutrients and inflammation. J. Clin. Investig..

[295] Chait A., den Hartigh L.J. (2020). Adipose Tissue Distribution, Inflammation and Its Metabolic Consequences, Including Diabetes and Cardiovascular Disease. Front. Cardiovasc. Med..

[296] Nguyen T.T., Ta Q.T.H., Nguyen T.T.D., Le T.T., Vo V.G. (2020). Role of Insulin Resistance in the Alzheimer’s Disease Progression. Neurochem. Res..

[297] Bahniwal M., Little J.P., Klegeris A. (2017). High Glucose Enhances Neurotoxicity and Inflammatory Cytokine Secretion by Stimulated Human Astrocytes. Curr. Alzheimer Res..

[298] Anjum I., Fayyaz M., Wajid A., Sohail W., Ali A. (2018). Does Obesity Increase the Risk of Dementia: A Literature Review. Cureus.

[299] Flores-Dorantes M.T., Díaz-López Y.E., Gutiérrez-Aguilar R. (2020). Environment and Gene Association With Obesity and Their Impact on Neurodegenerative and Neurodevelopmental Diseases. Front. Neurosci..

[300] Batatinha H.A., Biondo L.A., Lira F.S., Castell L.M., Rosa-Neto J.C. (2019). Nutrients, immune system, and exercise: Where will it take us?. Nutrition.

[301] Nieman D.C., Lila M.A., Gillitt N.D. (2019). Immunometabolism: A Multi-Omics Approach to Interpreting the Influence of Exercise and Diet on the Immune System. Annu. Rev. Food Sci. Technol..

[302] Simpson R.J., Lowder T.W., Spielmann G., Bigley A.B., LaVoy E.C., Kunz H. (2012). Exercise and the aging immune system. Ageing Res. Rev..

[303] Ostrowski K., Rohde T., Zacho M., Asp S., Pedersen B.K. (1998). Evidence that interleukin-6 is produced in human skeletal muscle during prolonged running. J. Physiol..

[304] Ma L., Zhang H., Yin Y.-L., Guo W.-Z., Ma Y.-Q., Wang Y.-B., Shu C., Dong L.-Q. (2016). Role of interleukin-6 to differentiate sepsis from non-infectious systemic inflammatory response syndrome. Cytokine.

[305] Febbraio M.A., Hiscock N., Sacchetti M., Fischer C.P., Pedersen B.K. (2004). Interleukin-6 Is a Novel Factor Mediating Glucose Homeostasis During Skeletal Muscle Contraction. Diabetes.

[306] Petersen E.W., Carey A.L., Sacchetti M., Steinberg G.R., Macaulay S.L., Febbraio M.A., Pedersen B.K. (2005). Acute IL-6 treatment increases fatty acid turnover in elderly humans in vivo and in tissue culture in vitro. Am. J. Physiol. Metab..

[307] Neto J.C., Silveira L.S. (2020). Endurance Exercise Mitigates Immunometabolic Adipose Tissue Disturbances in Cancer and Obesity. Int. J. Mol. Sci..

[308] Kwilasz A., Grace P., Serbedzija P., Maier S., Watkins L. (2015). The therapeutic potential of interleukin-10 in neuroimmune diseases. Neuropharmacology.

[309] Lobo-Silva D., Carriche G.M., Gil Castro A., Roque S., Saraiva M. (2016). Balancing the immune response in the brain: IL-10 and its regulation. J. Neuroinflamm..

[310] Littlefield A.M., Setti S.E., Priester C., Kohman R.A. (2015). Voluntary exercise attenuates LPS-induced reductions in neurogenesis and increases microglia expression of a proneurogenic phenotype in aged mice. J. Neuroinflamm..

[311] Rêgo M.L., Cabral D.A., Costa E.C., Fontes E.B. (2019). Physical Exercise for Individuals with Hypertension: It Is Time to Emphasize its Benefits on the Brain and Cognition. Clin. Med. Insights Cardiol..

[312] Di Liegro C.M., Schiera G., Proia P., Di Liegro I. (2019). Physical Activity and Brain Health. Genes.

[313] O’Toole P.W., Jeffery I.B. (2015). Gut microbiota and aging. Science.

[314] Vemuri R., Gundamaraju R., Shastri M.D., Shukla S.D., Kalpurath K., Ball M., Tristram S., Shankar E.M., Ahuja K., Eri R. (2018). Gut Microbial Changes, Interactions, and Their Implications on Human Lifecycle: An Ageing Perspective. BioMed Res. Int..

[315] Radjabzadeh D., Boer C.G., Beth S.A., Van Der Wal P., Jong J.C.K.-D., Jansen M.A.E., Konstantinov S.R., Peppelenbosch M.P., Hays J.P., Jaddoe V.W.V. (2020). Diversity, compositional and functional differences between gut microbiota of children and adults. Sci. Rep..

[316] Claesson M.J., Jeffery I.B., Conde S., Power S.E., O’Connor E.M., Cusack S., Harris H.M.B., Coakley M., Lakshminarayanan B., O’Sullivan O. (2012). Gut microbiota composition correlates with diet and health in the elderly. Nature.

[317] Jiang C., Li G., Huang P., Liu Z., Zhao B. (2017). The Gut Microbiota and Alzheimer’s Disease. J. Alzheimer's Dis..

[318] Bostanciklioğlu M. (2019). The role of gut microbiota in pathogenesis of Alzheimer’s disease. J. Appl. Microbiol..

[319] Talwar P., Kushwaha S., Gupta R., Agarwal R. (2019). Systemic Immune Dyshomeostasis Model and Pathways in Alzheimer’s Disease. Front. Aging Neurosci..

[320] Bonaz B., Bazin T., Pellissier S. (2018). The Vagus Nerve at the Interface of the Microbiota-Gut-Brain Axis. Front. Neurosci..

[321] Muller P.A., Schneeberger M., Matheis F., Wang P., Kerner Z., Ilanges A., Pellegrino K., Del Mármol J., Castro T.B.R., Furuichi M. (2020). Microbiota modulate sympathetic neurons via a gut–brain circuit. Nat. Cell Biol..

[322] Wang X., Sun G., Feng T., Zhang J., Huang X., Wang T., Xie Z., Chu X., Yang J., Wang H. (2019). Sodium oligomannate therapeutically remodels gut microbiota and suppresses gut bacterial amino acids-shaped neuroinflammation to inhibit Alzheimer’s disease progression. Cell Res..

[323] Rea K., Dinan T.G., Cryan J.F. (2016). The microbiome: A key regulator of stress and neuroinflammation. Neurobiol. Stress.

[324] Chen J., Buchanan J.B., Sparkman N.L., Godbout J.P., Freund G.G., Johnson R.W. (2008). Neuroinflammation and disruption in working memory in aged mice after acute stimulation of the peripheral innate immune system. Brain Behav. Immun..

[325] McManus R.M., Heneka M.T. (2017). Role of neuroinflammation in neurodegeneration: New insights. Alzheimer's Res. Ther..

[326] Carpanini S.M., Torvell M., Morgan B.P. (2019). Therapeutic Inhibition of the Complement System in Diseases of the Central Nervous System. Front. Immunol..

[327] Morgan B.P. (2018). Complement in the pathogenesis of Alzheimer’s disease. Semin. Immunopathol..

[328] Tenner A.J., Stevens B., Woodruff T.M. (2018). New tricks for an ancient system: Physiological and pathological roles of complement in the CNS. Mol. Immunol..

[329] Wu T., Dejanovic B., Gandham V.D., Gogineni A., Edmonds R., Schauer S., Srinivasan K., Huntley M.A., Wang Y., Wang T.M. (2019). Complement C3 is activated in human AD brain and is required for neurodegeneration in mouse models of amyloidosis and tauopathy. Cell Rep..

[330] Montagne A., Barnes S.R., Sweeney M.D., Halliday M.R., Sagare A.P., Zhao Z., Toga A.W., Jacobs R.E., Liu C.Y., Amezcua L. (2015). Blood-Brain Barrier Breakdown in the Aging Human Hippocampus. Neuron.

[331] Varatharaj A., Galea I. (2017). The blood-brain barrier in systemic inflammation. Brain Behav. Immun..

[332] Takeda S., Sato N., Morishita R. (2014). Systemic inflammation, blood-brain barrier vulnerability and cognitive/non-cognitive symptoms in Alzheimer disease: Relevance to pathogenesis and therapy. Front. Aging Neurosci..

[333] Jaeger L.B., Dohgu S., Sultana R., Lynch J.L., Owen J.B., Erickson M.A., Shah G.N., Price T.O., Fleegal-Demotta M.A., Butterfield D.A. (2009). Lipopolysaccharide alters the blood-brain barrier transport of amyloid beta protein: A mechanism for inflammation in the progression of Alzheimer’s disease. Brain Behav. Immun..

[334] Propson N.E., Roy E.R., Litvinchuk A., Köhl J., Zheng H. (2021). Endothelial C3a receptor mediates vascular inflammation and blood-brain barrier permeability during aging. J. Clin. Investig..

[335] Bhatia K., Ahmad S., Kindelin A., Ducruet A.F. (2021). Complement C3a receptor-mediated vascular dysfunction: A complex interplay between aging and neurodegeneration. J. Clin. Investig..

[336] Liddelow S.A., Guttenplan K.A., Clarke L.E., Bennett F.C., Bohlen C.J., Schirmer L., Bennett M.L., Münch A.E., Chung W.-S., Peterson T.C. (2017). Neurotoxic reactive astrocytes are induced by activated microglia. Nature.

[337] Perez-Nievas B.G., Serrano-Pozo A. (2018). Deciphering the Astrocyte Reaction in Alzheimer’s Disease. Front. Aging Neurosci..

[338] Seol Y., Ki S., Ryu H.L., Chung S., Lee J., Ryu H. (2020). How Microglia Manages Non-cell Autonomous Vicious Cycling of Aβ Toxicity in the Pathogenesis of AD. Front. Mol. Neurosci..

[339] Henstridge C.M., Hyman B.T., Spires-Jones T.L. (2019). Beyond the neuron–cellular interactions early in Alzheimer disease pathogenesis. Nat. Rev. Neurosci..

[340] Fullerton J.N., Gilroy D.W. (2016). Resolution of inflammation: A new therapeutic frontier. Nat. Rev. Drug Discov..

[341] Probert L. (2015). TNF and its receptors in the CNS: The essential, the desirable and the deleterious effects. Neuroscience.

[342] Steeland S., Vandenbroucke R.E.J. (2019). Choroid plexus tumor necrosis factor receptor 1: A new neuroinflammatory piece of the complex Alzheimer’s disease puzzle. Neural Regen Res..

[343] Belarbi K., Jopson T., Tweedie D., Arellano C., Luo W., Greig N.H., Rosi S. (2012). TNF-α protein synthesis inhibitor restores neuronal function and reverses cognitive deficits induced by chronic neuroinflammation. J. Neuroinflamm..

[344] Olmos G., Lladó J. (2014). Tumor necrosis factor alpha: A link between neuroinflammation and excitotoxicity. Mediat. Inflamm..

[345] Chang R., Yee K.L., Sumbria R.K. (2017). Tumor necrosis factor α Inhibition for Alzheimer’s Disease. J. Cent. Nerv. Syst. Dis..

[346] Paouri E., Tzara O., Kartalou G.-I., Zenelak S., Georgopoulos S. (2017). Peripheral Tumor Necrosis Factor-Alpha (TNF-α) Modulates Amyloid Pathology by Regulating Blood-Derived Immune Cells and Glial Response in the Brain of AD/TNF Transgenic Mice. J. Neurosci..

[347] Paouri E., Tzara O., Zenelak S., Georgopoulos S. (2017). Genetic Deletion of Tumor Necrosis Factor-α Attenuates Amyloid-β Production and Decreases Amyloid Plaque Formation and Glial Response in the 5XFAD Model of Alzheimer’s Disease. J. Alzheimer’s Dis..

[348] Steeland S., Gorlé N., VandenDriessche C., Balusu S., Brkic M., Van Cauwenberghe C., Van Imschoot G., Van Wonterghem E., De Rycke R., Kremer A. (2018). Counteracting the effects of TNF receptor-1 has therapeutic potential in Alzheimer’s disease. EMBO Mol. Med..

[349] Lapadula G., Marchesoni A., Armuzzi A., Blandizzi C., Caporali R., Chimenti S., Cimaz R., Cimino L., Gionchetti P., Girolomoni G. (2014). Adalimumab in the Treatment of Immune-Mediated Diseases. Int. J. Immunopathol. Pharmacol..

[350] Decourt B., Drumm-Gurnee D., Wilson J., Jacobson S., Belden C., Sirrel S., Ahmadi M., Shill H., Powell J., Walker A. (2017). Poor Safety and Tolerability Hamper Reaching a Potentially Therapeutic Dose in the Use of Thalidomide for Alzheimer’s disease: Results from a Double-Blind, Placebo-Controlled Trial. Curr. Alzheimer Res..

[351] Park J., Lee S.-Y., Shon J., Kim K., Lee H.J., Kim K.A., Lee B.-Y., Oh S.-H., Kim N.K., Kim O.J. (2019). Adalimumab improves cognitive impairment, exerts neuroprotective effects and attenuates neuroinflammation in an Aβ1-40-injected mouse model of Alzheimer’s disease. Cytotherapy.

[352] Anwar S., Rivest S. (2020). Alzheimer’s disease: Microglia targets and their modulation to promote amyloid phagocytosis and mitigate neuroinflammation. Expert Opin. Ther. Targets.

[353] Zhou M., Xu R., Kaelber D.C., Gurney M.E. (2020). Tumor Necrosis Factor (TNF) blocking agents are associated with lower risk for Alzheimer’s disease in patients with rheumatoid arthritis and psoriasis. PLoS ONE.

[354] Steed P.M., Tansey M.G., Zalevsky J., Zhukovsky E.A., DesJarlais J.R., Szymkowski D.E., Abbott C., Carmichael D., Chan C., Cherry L. (2003). Inactivation of TNF Signaling by Rationally Designed Dominant-Negative TNF Variants. Science.

[355] Cavanagh C., Tse Y.C., Nguyen H.-B., Krantic S., Breitner J.C., Quirion R., Wong T.P. (2016). Inhibiting tumor necrosis factor-α before amyloidosis prevents synaptic deficits in an Alzheimer’s disease model. Neurobiol. Aging.

[356] MacPherson K.P., Sompol P., Kannarkat G.T., Chang J., Sniffen L., Wildner M.E., Norris C.M., Tansey M.G. (2017). Peripheral administration of the soluble TNF inhibitor XPro1595 modifies brain immune cell profiles, decreases beta-amyloid plaque load, and rescues impaired long-term potentiation in 5xFAD mice. Neurobiol. Dis..

[357] McAlpine F.E., Lee J.K., Harms A.S., Ruhn K.A., Blurton-Jones M., Hong J., Das P., Golde T.E., LaFerla F.M., Oddo S. (2009). Inhibition of soluble TNF signaling in a mouse model of Alzheimer’s disease prevents pre-plaque amyloid-associated neuropathology. Neurobiol. Dis..

[358] Sama D.M., Mohmmad Abdul H., Furman J.L., Artiushin I.A., Szymkowski D.E., Scheff S.W., Norris C.M. (2012). Inhibition of soluble tumor necrosis factor ameliorates synaptic alterations and Ca2+ dysregulation in aged rats. PLoS ONE.

[359] Yiannopoulou K.G., Papageorgiou S.G. (2020). Current and Future Treatments in Alzheimer Disease: An Update. J. Cent. Nerv. Syst. Dis..

[360] Jung Y.J., Tweedie D., Scerba M.T., Greig N.H. (2019). Neuroinflammation as a Factor of Neurodegenerative Disease: Thalidomide Analogs as Treatments. Front. Cell Dev. Biol..

[361] He P., Cheng X., Staufenbiel M., Li R., Shen Y. (2013). Long-Term Treatment of Thalidomide Ameliorates Amyloid-Like Pathology through Inhibition of β-Secretase in a Mouse Model of Alzheimer’s Disease. PLoS ONE.

[362] Decourt B., Wilson J., Ritter A., Dardis C., DiFilippo F.P., Zhuang X., Cordes D., Lee G., Fulkerson N.D., Rose T.S. (2020). MCLENA-1: A Phase II Clinical Trial for the Assessment of Safety, Tolerability, and Efficacy of Lenalidomide in Patients with Mild Cognitive Impairment Due to Alzheimer’s Disease. Open Access J. Clin. Trials.

[363] Tweedie D., Ferguson R.A., Fishman K., Frankola K.A., Van Praag H., Holloway H.W., Luo W., Li Y., Caracciolo L., Russo I. (2012). Tumor necrosis factor-α synthesis inhibitor 3,6’-dithiothalidomide attenuates markers of inflammation, Alzheimer pathology and behavioral deficits in animal models of neuroinflammation and Alzheimer’s disease. J. Neuroinflamm..

[364] Lin C.T., Lecca D., Yang L.Y., Luo W., Scerba M.T., Tweedie D., Huang P.S., Jung Y.J., Kim D.S., Yang C.H. (2020). 3,6’-dithiopomalidomide reduces neural loss, inflammation, behavioral deficits in brain injury and microglial activation. Elife.

[365] Russo I., Caracciolo L., Tweedie D., Choi S.-H., Greig N.H., Barlati S., Bosetti F. (2012). 3,6′-Dithiothalidomide, a new TNF-α synthesis inhibitor, attenuates the effect of Aβ1-42 intracerebroventricular injection on hippocampal neurogenesis and memory deficit. J. Neurochem..

[366] Gabbita S.P., Srivastava M.K., Eslami P., Johnson M.F., Kobritz N.K., Tweedie D., Greig N.H., Zemlan F.P., Sharma S.P., Harris-White M.E. (2012). Early intervention with a small molecule inhibitor for tumor necrosis factor-α prevents cognitive deficits in a triple transgenic mouse model of Alzheimer’s disease. J. Neuroinflamm..

[367] Kirkland J.L., Tchkonia T., Zhu Y., Niedernhofer L.J., Robbins P.D. (2017). The Clinical Potential of Senolytic Drugs. J. Am. Geriatr. Soc..

[368] Kirkland J.L., Tchkonia T. (2017). Cellular senescence: A translational perspective. eBioMedicine.

[369] Zhu Y., Doornebal E.J., Pirtskhalava T., Giorgadze N., Wentworth M., Fuhrmann-Stroissnigg H., Niedernhofer L.J., Robbins P.D., Tchkonia T., Kirkland J.L. (2017). New agents that target senescent cells: The flavone, fisetin, and the BCL-XL inhibitors, A1331852 and A1155463. Aging.

[370] Baker D.J., Petersen R.C. (2018). Cellular senescence in brain aging and neurodegenerative diseases: Evidence and perspectives. J. Clin. Investig..

[371] Farr J.N., Fraser D.G., Wang H., Jaehn K., Ogrodnik M.B., Weivoda M.M., Drake M.T., Tchkonia T., Lebrasseur N.K., Kirkland J.L. (2016). Identification of Senescent Cells in the Bone Microenvironment. J. Bone Miner. Res..

[372] Jurk D., Wilson C., Passos J.F., Oakley F., Correia-Melo C., Greaves L., Saretzki G., Fox C., Lawless C., Anderson R. (2014). Chronic inflammation induces telomere dysfunction and accelerates ageing in mice. Nat. Commun..

[373] Sabogal-Guaqueta A.M., Munoz-Manco J.I., Ramirez-Pineda J.R., Lamprea-Rodriguez M., Osorio E., Cardona-Gomez G.P. (2015). The flavonoid quercetin ameliorates Alzheimer’s disease pathology and protects cognitive and emotional function in aged triple transgenic Alzheimer’s disease model mice. Neuropharmacology.

[374] Kirkland J.L., Tchkonia T. (2020). Senolytic drugs: From discovery to translation. J. Intern. Med..

[375] Wissler Gerdes E.O., Zhu Y., Weigand B.M., Tripathi U., Burns T.C., Tchkonia T., Kirkland J.L. (2020). Cellular senescence in aging and age-related diseases: Implications for neurodegenerative diseases. Int. Rev. Neurobiol..

[376] Yousefzadeh M.J., Zhu Y., McGowan S.J., Angelini L., Fuhrmann-Stroissnigg H., Xu M., Ling Y.Y., Melos K.I., Pirtskhalava T., Inman C.L. (2018). Fisetin is a senotherapeutic that extends health and lifespan. EBioMedicine.

[377] Garbarino V.R., Tran S., E Glassman J., Kirkland J.L., Musi N., Seshadri S., E Orr M. (2020). A head-to-head comparison between senolytic therapies, dasatinib plus quercetin and fisetin, indicates sex- and genotype-specific differences in translationally relevant outcomes. Alzheimer’s Dement..

[378] Jiang H., Gong T., Zhou R. (2020). The strategies of targeting the NLRP3 inflammasome to treat inflammatory diseases. Adv. Immunol..

[379] Chauhan D., Walle L.V., Lamkanfi M. (2020). Therapeutic modulation of inflammasome pathways. Immunol. Rev..

[380] Zheng D., Liwinski T., Elinav E. (2020). Inflammasome activation and regulation: Toward a better understanding of complex mechanisms. Cell Discov..

[381] Goldberg E.L., Dixit V.D. (2015). Drivers of age-related inflammation and strategies for healthspan extension. Immunol Rev..

[382] El-Sharkawy L.Y., Brough D., Freeman S. (2020). Inhibiting the NLRP3 Inflammasome. Molecules.

[383] Coll R.C., Robertson A.A.B., Chae J.J., Higgins S.C., Muñoz-Planillo R., Inserra M.C., Vetter I., Dungan L.S., Monks B.G., Stutz A. (2015). A small-molecule inhibitor of the NLRP3 inflammasome for the treatment of inflammatory diseases. Nat. Med..

[384] Coll R.C., Hill J.R., Day C.J., Zamoshnikova A., Boucher D., Massey N.L., Chitty J.L., Fraser J.A., Jennings M.P., Robertson A.A.B. (2019). MCC950 directly targets the NLRP3 ATP-hydrolysis motif for inflammasome inhibition. Nat. Chem. Biol..

[385] Jiang M., Li R., Lyu J., Li X., Wang W., Wang Z., Sheng H., Zhang W., Karhausen J., Yang W. (2020). MCC950, a selective NLPR3 inflammasome inhibitor, improves neurologic function and survival after cardiac arrest and resuscitation. J. Neuroinflamm..

[386] Dempsey C., Rubio Araiz A., Bryson K.J., Finucane O., Larkin C., Mills E.L., Robertson A.A., Cooper M.A., O’Neill L.A., Lynch M.A. (2017). Inhibiting the NLRP3 inflammasome with MCC950 promotes non-phlogistic clearance of amyloid-β and cognitive function in APP/PS1 mice. Brain Behav. Immun..

[387] Riella L.V., Paterson A.M., Sharpe A.H., Chandraker A. (2012). Role of the PD-1 Pathway in the Immune Response. Arab. Archaeol. Epigr..

[388] Zhao J., Ji R.-R. (2019). Anti-PD-1 treatment as a neurotherapy to enhance neuronal excitability, synaptic plasticity and memory. bioRxiv.

[389] Laumet G., Ma J., Robison A.J., Kumari S., Heijnen C.J., Kavelaars A. (2019). T Cells as an Emerging Target for Chronic Pain Therapy. Front. Mol. Neurosci..

[390] Chamoto K., Al-Habsi M., Honjo T. (2017). Role of PD-1 in Immunity and Diseases. Curr. Top. Microbiol. Immunol..

[391] Curdy N., Lanvin O., Laurent C., Fournié J.-J., Franchini D.-M. (2019). Regulatory Mechanisms of Inhibitory Immune Checkpoint Receptors Expression. Trends Cell Biol..

[392] Baruch K., Rosenzweig N., Kertser A., Deczkowska A., Sharif A.M., Spinrad A., Tsitsou-Kampeli A., Sarel A., Cahalon L., Schwartz M. (2015). Breaking immune tolerance by targeting Foxp3+ regulatory T cells mitigates Alzheimer’s disease pathology. Nat. Commun..

[393] Santarpia M., González-Cao M., Viteri S., Karachaliou N., Altavilla G., Rosell R. (2015). Programmed cell death protein-1/programmed cell death ligand-1 pathway inhibition and predictive biomarkers: Understanding transforming growth factor-beta role. Transl. Lung Cancer Res..

[394] Schwartz M., Arad M., Ben-Yehuda H. (2019). Potential immunotherapy for Alzheimer disease and age-related dementia. Dialog. Clin. Neurosci.

[395] Munafò A., Burgaletto C., Di Benedetto G., Di Mauro M., Di Mauro R., Bernardini R., Cantarella G. (2020). Repositioning of Immunomodulators: A Ray of Hope for Alzheimer’s Disease?. Front. Neurosci..

[396] Baruch K., Deczkowska A., Rosenzweig N., Tsitsou-Kampeli A., Sharif A.M., Matcovitch-Natan O., Kertser A., David E., Amit I., Schwartz M. (2016). PD-1 immune checkpoint blockade reduces pathology and improves memory in mouse models of Alzheimer’s disease. Nat. Med..

[397] Rogers N.K., Romero C., Sanmartín C.D., Ponce D.P., Salech F., López M.N., Gleisner A., Tempio F., Behrens M.I. (2020). Inverse Relationship Between Alzheimer’s Disease and Cancer: How Immune Checkpoints Might Explain the Mechanisms Underlying Age-Related Diseases. J. Alzheimer’s Dis..

[398] Rosenzweig N., Dvir-Szternfeld R., Tsitsou-Kampeli A., Keren-Shaul H., Ben-Yehuda H., Weill-Raynal P., Cahalon L., Kertser A., Baruch K., Amit I. (2019). PD-1/PD-L1 checkpoint blockade harnesses monocyte-derived macrophages to combat cognitive impairment in a tauopathy mouse model. Nat. Commun..

[399] Latta-Mahieu M., Elmer B., Bretteville A., Wang Y., Lopez-Grancha M., Goniot P., Moindrot N., Ferrari P., Blanc V., Schussler N. (2017). Systemic immune-checkpoint blockade with anti-PD1 antibodies does not alter cerebral amyloid-β burden in several amyloid transgenic mouse models. Glia.

[400] Obst J., Mancuso R., Simon E., Gomez-Nicola D. (2018). PD-1 deficiency is not sufficient to induce myeloid mobilization to the brain or alter the inflammatory profile during chronic neurodegeneration. Brain Behav. Immun..

[401] Li S., Hayden E.Y., Garcia V.J., Fuchs D.-T., Sheyn J., Daley D.A., Rentsendorj A., Torbati T., Black K.L., Rutishauser U. (2020). Activated bone marrow-derived macrophages eradicate Alzheimer’s-related Aβ42 Oligomers and protect synapses. Front. Immunol..

[402] van de Donk N.W.C.J. (2018). Immunomodulatory effects of CD38-targeting antibodies. Immunol. Lett..

[403] Guerreiro S., Privat A.-L., Bressac L., Toulorge D. (2020). CD38 in Neurodegeneration and Neuroinflammation. Cells.

[404] Blacher E., Dadali T., Bespalko A., Msc V.J.H., Grimm M.O.W., Hartmann T., Lund F.E., Stein R., Levy A. (2015). Alzheimer’s disease pathology is attenuated in a CD38-deficient mouse model. Ann. Neurol..

[405] Zhao L. (2019). CD33 in Alzheimer’s Disease—Biology, Pathogenesis, and Therapeutics: A Mini-Review. Gerontology.

[406] Malik M., Simpson J.F., Parikh I., Wilfred B.R., Fardo D.W., Nelson P.T., Estus S. (2013). CD33 Alzheimer’s Risk-Altering Polymorphism, CD33 Expression, and Exon 2 Splicing. J. Neurosci..

[407] Griciuc A., Serrano-Pozo A., Parrado A.R., Lesinski A.N., Asselin C.N., Mullin K., Hooli B., Choi S.H., Hyman B.T., Tanzi R.E. (2013). Alzheimer’s Disease Risk Gene CD33 Inhibits Microglial Uptake of Amyloid Beta. Neuron.

[408] Miles L.A., Hermans S.J., Crespi G.A., Gooi J.H., Doughty L., Nero T.L., Markulić J., Ebneth A., Wroblowski B., Oehlrich D. (2019). Small Molecule Binding to Alzheimer Risk Factor CD33 Promotes Aβ Phagocytosis. iScience.

[409] Vom Berg J., Prokop S., Miller K.R., Obst J., Kälin R.E., Lopategui-Cabezas I., Wegner A., Mair F., Schipke C.G., Peters O. (2012). Inhibition of IL-12/IL-23 signaling reduces Alzheimer’s disease-like pathology and cognitive decline. Nat. Med..

[410] Town T., Vendrame M., Patel A., Poetter D., DelleDonne A., Mori T., Smeed R., Crawford F., Klein T., Tan J. (2002). Reduced Th1 and enhanced Th2 immunity after immunization with Alzheimer’s beta-amyloid(1-42). J. Neuroimmunol..

[411] Hu W.T., Holtzman D.M., Fagan A.M., Shaw L.M., Perrin R., Arnold S.E., Grossman M., Xiong C., Craig-Schapiro R., Clark C.M. (2012). Plasma multianalyte profiling in mild cognitive impairment and Alzheimer disease. Neurology.

[412] Teng M.W.L., Bowman E.P., McElwee J.J., Smyth M.J., Casanova J.-L., Cooper A.M., Cua D.J. (2015). IL-12 and IL-23 cytokines: From discovery to targeted therapies for immune-mediated inflammatory diseases. Nat. Med..

[413] Scott L.J. (2013). Glatiramer acetate: A review of its use in patients with relapsing-remitting multiple sclerosis and in delaying the onset of clinically definite multiple sclerosis. CNS Drugs.

[414] Arnon R., Aharoni R. (2019). Glatiramer Acetate: From Bench to Bed and Back. ISR Med. Assoc. J..

[415] Chen L., Yao Y., Wei C., Sun Y., Ma X., Zhang R., Xu X., Hao J. (2015). T cell immunity to glatiramer acetate ameliorates cognitive deficits induced by chronic cerebral hypoperfusion by modulating the microenvironment. Sci. Rep..

[416] Vieira P.L., Heystek H.C., Wormmeester J., Wierenga E.A., Kapsenberg M.L. (2003). Glatiramer Acetate (Copolymer-1, Copaxone) Promotes Th2 Cell Development and Increased IL-10 Production Through Modulation of Dendritic Cells. J. Immunol..

[417] Lalive P.H., Neuhaus O., Benkhoucha M., Burger D., Hohlfeld R., Zamvil S.S., Weber M.S. (2011). Glatiramer acetate in the treatment of multiple sclerosis: Emerging concepts regarding its mechanism of action. CNS Drugs.

[418] Koronyo Y., Salumbides B.C., Sheyn J., Pelissier L., Li S., Ljubimov V., Moyseyev M., Daley D., Fuchs D.-T., Pham M. (2015). Therapeutic effects of glatiramer acetate and grafted CD115+monocytes in a mouse model of Alzheimer’s disease. Brain.

[419] Butovsky O., Koronyo-Hamaoui M., Kunis G., Ophir E., Landa G., Cohen H., Schwartz M. (2006). Glatiramer acetate fights against Alzheimer’s disease by inducing dendritic-like microglia expressing insulin-like growth factor 1. Proc. Natl. Acad. Sci. USA.

[420] Frenkel D., Maron R., Burt D.S., Weiner H.L. (2005). Nasal vaccination with a proteosome-based adjuvant and glatiramer acetate clears beta-amyloid in a mouse model of Alzheimer disease. J. Clin. Investig..

[421] Bakalash S., Pham M., Koronyo Y., Salumbides B.C., Kramerov A., Seidenberg H., Berel D., Black K.L., Koronyo-Hamaoui M. (2011). Egr1 Expression Is Induced Following Glatiramer Acetate Immunotherapy in Rodent Models of Glaucoma and Alzheimer’s Disease. Investig. Opthalmology Vis. Sci..

[422] Khanna A.K. (2000). MECHANISM OF THE COMBINATION IMMUNOSUPPRESSIVE EFFECTS OF RAPAMYCIN WITH EITHER CYCLOSPORINE OR TACROLIMUS. Transplantation.

[423] Lee R.K.K., Knapp S., Wurtman R.J. (1999). Prostaglandin E2 Stimulates Amyloid Precursor Protein Gene Expression: Inhibition by Immunosuppressants. J. Neurosci..

[424] Rojanathammanee L., Floden A.M., Manocha G.D., Combs C.K. (2015). Attenuation of microglial activation in a mouse model of Alzheimer’s disease via NFAT inhibition. J. Neuroinflamm..

[425] Rozkalne A., Hyman B.T., Spires-Jones T.L. (2011). Calcineurin inhibition with FK506 ameliorates dendritic spine density deficits in plaque-bearing Alzheimer model mice. Neurobiol. Dis..

[426] Kumar A., Singh N. (2017). Calcineurin inhibitors improve memory loss and neuropathological changes in mouse model of dementia. Pharmacol. Biochem. Behav..

[427] Alam J., Blackburn K., Patrick D. (2017). Neflamapimod: Clinical Phase 2b-Ready Oral Small Molecule Inhibitor of p38α to Reverse Synaptic Dysfunction in Early Alzheimer’s Disease. J. Prev. Alzheimer's Dis..

[428] Alam J.J. (2015). Selective Brain-Targeted Antagonism of p38 MAPKα Reduces Hippocampal IL-1β Levels and Improves Morris Water Maze Performance in Aged Rats. J. Alzheimer’s Dis..

[429] Fonseca M.I., Ager R.R., Chu S.-H., Yazan O., Sanderson S.D., LaFerla F.M., Taylor S.M., Woodruff T.M., Tenner A.J. (2009). Treatment with a C5aR Antagonist Decreases Pathology and Enhances Behavioral Performance in Murine Models of Alzheimer’s Disease. J. Immunol..

[430] Hong S., Beja-Glasser V.F., Nfonoyim B.M., Frouin A., Li S., Ramakrishnan S., Merry K.M., Shi Q., Rosenthal A., Barres B.A. (2016). Complement and microglia mediate early synapse loss in Alzheimer mouse models. Science.

[431] Lansita J.A., Mease K.M., Qiu H., Yednock T., Sankaranarayanan S., Kramer S. (2017). Nonclinical Development of ANX005: A Humanized Anti-C1q Antibody for Treatment of Autoimmune and Neurodegenerative Diseases. Int. J. Toxicol..

[432] Mathieu M.-C., Sawyer N., Greig G.M., Hamel M., Kargman S., Ducharme Y., Lau C.K., Friesen R.W., O’Neill G.P., Gervais F.G. (2005). The C3a receptor antagonist SB 290157 has agonist activity. Immunol. Lett..

[433] Porrini V., Lanzillotta A., Branca C., Benarese M., Parrella E., Lorenzini L., Calza L., Flaibani R., Spano P., Imbimbo B. (2015). CHF5074 (CSP-1103) induces microglia alternative activation in plaque-free Tg2576 mice and primary glial cultures exposed to beta-amyloid. Neuroscience.

[434] Hwangbo D.-S., Lee H.-Y., Abozaid L.S., Min K.-J. (2020). Mechanisms of Lifespan Regulation by Calorie Restriction and Intermittent Fasting in Model Organisms. Nutrients.

[435] Dorling J.L., Martin C.K., Redman L.M. (2020). Calorie restriction for enhanced longevity: The role of novel dietary strategies in the present obesogenic environment. Ageing Res. Rev..

[436] Chung H.Y., Kim H.J., Kim J.W., Yu B.P. (2001). The inflammation hypothesis of aging: Molecular modulation by calorie restriction. Ann. N. Y. Acad. Sci..

[437] Di Francesco A., Di Germanio C., Bernier M., de Cabo R. (2018). A time to fast. Science.

[438] Wang Y.-W., He S.-J., Feng X., Cheng J., Luo Y.-T., Tian L., Huang Q. (2017). Metformin: A review of its potential indications. Drug Des. Dev. Ther..

[439] Vieira R., Souto S.B., Sánchez-López E., Machado A.L., Severino P., Jose S., Santini A., Fortuna A., García M.L., Silva A.M. (2019). Sugar-Lowering Drugs for Type 2 Diabetes Mellitus and Metabolic Syndrome—Review of Classical and New Compounds: Part-I. Pharmaceuticals.

[440] Partridge L., Piper M.D., Mair W. (2005). Dietary restriction in Drosophila. Mech. Ageing Dev..

[441] Masoro E.J. (2000). Caloric restriction and aging: An update. Exp. Gerontol..

[442] Onken B., Driscoll M. (2010). Metformin induces a dietary restriction-like state and the oxidative stress response to extend C. elegans Healthspan via AMPK, LKB1, and SKN-1. PLoS ONE.

[443] Bharath L.P., Agrawal M., McCambridge G., Nicholas D.A., Hasturk H., Liu J., Jiang K., Liu R., Guo Z., Deeney J. (2020). Metformin Enhances Autophagy and Normalizes Mitochondrial Function to Alleviate Aging-Associated Inflammation. Cell Metab..

[444] Kang E.B., Koo J.H., Jang Y.C., Yang C.H., Lee Y., Cosio-Lima L.M., Cho J.Y. (2016). Neuroprotective Effects of Endurance Exercise Against High-Fat Diet-Induced Hippocampal Neuroinflammation. J. Neuroendocrinol..

[445] Park J., Cheon W., Kim K. (2020). Effects of Long-Term Endurance Exercise and Lithium Treatment on Neuroprotective Factors in Hippocampus of Obese Rats. Int. J. Environ. Res. Public Heal..

[446] Onyango I.G., Lu J., Rodova M., Lezi E., Crafter A.B., Swerdlow R.H. (2010). Regulation of neuron mitochondrial biogenesis and relevance to brain health. Biochim. Biophys. Acta (BBA) Mol. Basis Dis..

[447] Radak Z., Suzuki K., Higuchi M., Balogh L., Boldogh I., Koltai E. (2016). Physical exercise, reactive oxygen species and neuroprotection. Free Radic. Biol. Med..

[448] Intlekofer K.A., Cotman C.W. (2013). Exercise counteracts declining hippocampal function in aging and Alzheimer’s disease. Neurobiol. Dis..

[449] Dalle S., Rossmeislova L., Koppo K. (2017). The Role of Inflammation in Age-Related Sarcopenia. Front. Physiol..

[450] Nilsson M.I., Bourgeois J.M., Nederveen J.P., Leite M.R., Hettinga B.P., Bujak A.L., May L., Lin E., Crozier M., Rusiecki D.R. (2019). Lifelong aerobic exercise protects against inflammaging and cancer. PLoS ONE.

[451] Hardeland R. (2018). Melatonin and inflammation-Story of a double-edged blade. J. Pineal Res..

[452] Hardeland R. (2019). Aging, Melatonin, and the Pro- and Anti-Inflammatory Networks. Int. J. Mol. Sci..

[453] Marchal J., Pifferi F., Aujard F. (2013). Resveratrol in mammals: Effects on aging biomarkers, age-related diseases, and life span. Ann. N. Y. Acad. Sci..

[454] Alcaín F.J., Villalba J.M. (2009). Sirtuin activators. Expert. Opin. Ther. Pat..

[455] Zhu X., Liu Q., Wang M., Liang M., Yang X., Xu X., Zou H., Qiu J. (2011). Activation of Sirt1 by Resveratrol Inhibits TNF-α Induced Inflammation in Fibroblasts. PLoS ONE.

[456] Arbo B.D., André-Miral C., Nasre-Nasser R.G., Schimith L.E., Santos M.G., Costa-Silva D., Muccillo-Baisch A.L., Hort M.A. (2020). Resveratrol Derivatives as Potential Treatments for Alzheimer’s and Parkinson’s Disease. Front. Aging Neurosci..

[457] Moussa C., Hebron M., Huang X., Ahn J., Rissman R.A., Aisen P.S., Turner R.S. (2017). Resveratrol regulates neuro-inflammation and induces adaptive immunity in Alzheimer’s disease. J. Neuroinflamm..

[458] Turner R.S., Thomas R.G., Craft S., Van Dyck C.H., Mintzer J., Reynolds B.A., Brewer J.B., Rissman R.A., Raman R., Aisen P.S. (2015). A randomized, double-blind, placebo-controlled trial of resveratrol for Alzheimer disease. Neurology.

[459] Witte A.V., Kerti L., Margulies D.S., Flöel A. (2014). Effects of resveratrol on memory performance, hippocampal functional connectivity, and glucose metabolism in healthy older adults. J. Neurosci..

[460] Jiao F., Gong Z. (2020). The Beneficial Roles of SIRT1 in Neuroinflammation-Related Diseases. Oxid. Med. Cell Longev..

[461] Cannizzo E.S., Clement C.C., Sahu R., Follo C., Santambrogio L. (2011). Oxidative stress, inflamm-aging and immunosenescence. J. Proteom..

[462] Bachmann M.C., Bellalta S., Basoalto R., Gómez-Valenzuela F., Jalil Y., Lépez M., Matamoros A., Von Bernhardi R. (2020). The Challenge by Multiple Environmental and Biological Factors Induce Inflammation in Aging: Their Role in the Promotion of Chronic Disease. Front. Immunol..

[463] Chelombitko M.A. (2018). Role of Reactive Oxygen Species in Inflammation: A Minireview. Mosc. Univ. Biol. Sci. Bull..

[464] Jiang Q., Yin J., Chen J., Ma X., Wu M., Liu G., Yao K., Tan B., Yin Y. (2020). Mitochondria-Targeted Antioxidants: A Step towards Disease Treatment. Oxidative Med. Cell. Longev..

[465] Fujimoto C., Yamasoba T. (2019). Mitochondria-Targeted Antioxidants for Treatment of Hearing Loss: A Systematic Review. Antioxidants.

[466] Oyewole A.O., Birch-Machin M.A. (2015). Mitochondria-targeted antioxidants. FASEB J..

[467] Fang Y., Hu X.H., Jia Z.G., Xu M.H., Guo Z.Y., Gao F.H. (2012). Tiron protects against UVB-induced senescence-like characteristics in human dermal fibroblasts by the inhibition of superoxide anion production and glutathione depletion. Australas. J. Dermatol..

[468] Piermarocchi S., Saviano S., Parisi V., Tedeschi M., Panozzo G., Scarpa G., Boschi G., Giudice G.L., Sartore M., For The Carmis Study Group (2012). Carotenoids in Age-Related Maculopathy Italian Study (CARMIS): Two-Year Results of a Randomized Study. Eur. J. Ophthalmol..

[469] Wu W., Wang X., Xiang Q., Meng X., Peng Y., Du N., Liu Z., Sun Q., Wang C., Liu X. (2013). Astaxanthin alleviates brain aging in rats by attenuating oxidative stress and increasing BDNF levels. Food Funct..

[470] Gill H.S., Rutherfurd K.J., Cross M.L., Gopal P.K. (2001). Enhancement of immunity in the elderly by dietary supplementation with the probiotic Bifidobacterium lactis HN019. Am. J. Clin. Nutr..

[471] Dong H., Rowland I., Thomas L.V., Yaqoob P. (2013). Immunomodulatory effects of a probiotic drink containing Lactobacillus casei Shirota in healthy older volunteers. Eur. J. Nutr..

[472] Vulevic J., Drakoularakou A., Yaqoob P., Tzortzis G., Gibson G.R. (2008). Modulation of the fecal microflora profile and immune function by a novel trans-galactooligosaccharide mixture (B-GOS) in healthy elderly volunteers. Am. J. Clin. Nutr..

[473] Frasca D., Blomberg B.B. (2016). Inflammaging decreases adaptive and innate immune responses in mice and humans. Biogerontology.

[474] Man A.W.C., Zhou Y., Xia N., Li H. (2020). Involvement of Gut Microbiota, Microbial Metabolites and Interaction with Polyphenol in Host Immunometabolism. Nutrients.

[475] Angelucci F., Cechova K., Amlerova J., Hort J. (2019). Antibiotics, gut microbiota, and Alzheimer’s disease. J. Neuroinflamm..

[476] He Y., Li B., Sun D., Chen S. (2020). Gut Microbiota: Implications in Alzheimer’s Disease. J. Clin. Med..

[477] Haran J.P., Bhattarai S.K., Foley S.E., Dutta P., Ward D.V., Bucci V., McCormick B.A. (2019). Alzheimer’s Disease Microbiome Is Associated with Dysregulation of the Anti-Inflammatory P-Glycoprotein Pathway. mBio.

[478] Zhu F., Li C., Chu F., Tian X., Zhu J. (2020). Target Dysbiosis of Gut Microbes as a Future Therapeutic Manipulation in Alzheimer’s Disease. Front. Aging Neurosci..

[479] Sharma R., Padwad Y. (2020). Probiotic bacteria as modulators of cellular senescence: Emerging concepts and opportunities. Gut Microbes.

[480] Shabbir U., Arshad M., Sameen A., Oh D.-H. (2021). Crosstalk between Gut and Brain in Alzheimer’s Disease: The Role of Gut Microbiota Modulation Strategies. Nutrients.

[481] Kesika P., Suganthy N., Sivamaruthi B.S., Chaiyasut C. (2021). Role of gut-brain axis, gut microbial composition, and probiotic intervention in Alzheimer’s disease. Life Sci..

[482] Bonfili L., Cecarini V., Gogoi O., Gong C., Cuccioloni M., Angeletti M., Rossi G., Eleuteri A.M. (2020). Microbiota modulation as preventative and therapeutic approach in Alzheimer’s disease. FEBS J..

[483] Liu S., Gao J., Zhu M., Liu K., Zhang H.-L. (2020). Gut Microbiota and Dysbiosis in Alzheimer’s Disease: Implications for Pathogenesis and Treatment. Mol. Neurobiol..

[484] Portis S.M., Chaput D., Burroughs B., Hudson C., Sanberg P.R., Bickford P.C. (2020). Effects of nutraceutical intervention on serum proteins in aged rats. GeroScience.

[485] Sadhukhan P., Saha S., Dutta S., Mahalanobish S., Sil P.C. (2018). Nutraceuticals: An emerging therapeutic approach against the pathogenesis of Alzheimer’s disease. Pharmacol. Res..

[486] Calfio C., Gonzalez A., Singh S.K., Rojo L.E., Maccioni R.B. (2020). The Emerging Role of Nutraceuticals and Phytochemicals in the Prevention and Treatment of Alzheimer’s Disease. J. Alzheimer’s Dis..

[487] Vaiserman A., Koliada A., Lushchak O. (2020). Neuroinflammation in pathogenesis of Alzheimer’s disease: Phytochemicals as potential therapeutics. Mech. Ageing Dev..

[488] Flowers A., Lee J.-Y., Acosta S., Hudson C., Small B., Sanberg C.D., Bickford P.C., Grimmig B. (2015). NT-020 treatment reduces inflammation and augments Nrf-2 and Wnt signaling in aged rats. J. Neuroinflamm..

[489] Alpert P.T. (2017). The Role of Vitamins and Minerals on the Immune System. Home Heal. Care Manag. Pr..

[490] Mora J.R., Iwata M., von Andrian U.H. (2008). Vitamin effects on the immune system: Vitamins A and D take centre stage. Nat. Rev. Immunol..

[491] Maggini S., Pierre A., Calder P.C. (2018). Immune Function and Micronutrient Requirements Change over the Life Course. Nutrients.

[492] Abiri B., Vafa M. (2020). Micronutrients that Affect Immunosenescence. Adv. Exp. Med. Biol..

[493] Meydani S.N., Meydani M., Blumberg J.B., Leka L.S., Siber G., Loszewski R., Thompson C., Pedrosa M.C., Diamond R.D., Stollar B.D. (1997). Vitamin E supplementation and in vivo immune response in healthy elderly subjects. A randomized controlled trial. JAMA.

[494] De la Fuente M., Hernanz A., Guayerbas N., Victor V.M., Arnalich F. (2008). Vitamin E ingestion improves several immune functions in elderly men and women. Free Radic Res..

[495] Pallast E.G., Schouten E.G., De Waart F.G., Fonk H.C., Doekes G., Von Blomberg B.M., Kok F.J. (1999). Effect of 50- and 100-mg vitamin E supplements on cellular immune function in noninstitutionalized elderly persons. Am. J. Clin. Nutr..

[496] Tan B.L., Norhaizan M.E., Liew W.-P.-P., Rahman H.S. (2018). Antioxidant and Oxidative Stress: A Mutual Interplay in Age-Related Diseases. Front. Pharmacol..

[497] Tan B.L., Norhaizan M.E. (2019). Carotenoids: How Effective Are They to Prevent Age-Related Diseases?. Molecules.

[498] Monacelli F., Acquarone E., Giannotti C., Borghi R., Nencioni A. (2017). Vitamin C, Aging and Alzheimer’s Disease. Nutrients.

[499] Maggini S., Wintergerst E.S., Beveridge S., Hornig D.H. (2007). Selected vitamins and trace elements support immune function by strengthening epithelial barriers and cellular and humoral immune responses. Br. J. Nutr..

[500] Zhitkovich A. (2020). Nuclear and Cytoplasmic Functions of Vitamin C. Chem. Res. Toxicol..

[501] Hemilae H. (2017). Vitamin C and infections. Nutrients.

[502] Carr A.C., Maggini S. (2017). Vitamin C and Immune Function. Nutrients.

[503] Tanaka M., Muto N., Gohda E., Yamamoto I. (1994). Enhancement by Ascorbic Acid 2-Glucoside or Repeated Additions of Ascorbate of Mitogen-Induced IgM and IgG Productions by Human Peripheral Blood Lymphocytes. Jpn. J. Pharmacol..

[504] Cabrera Á.J.R. (2015). Zinc, aging, and immunosenescence: An overview. Pathobiol. Aging Age-Related Dis..

[505] Jarosz M., Olbert M., Wyszogrodzka G., Młyniec K., Librowski T. (2017). Antioxidant and anti-inflammatory effects of zinc. Zinc-dependent NF-κB signaling. Inflammopharmacology.

[506] Wong C.P., Ho E. (2012). Zinc and its role in age-related inflammation and immune dysfunction. Mol. Nutr. Food Res..

[507] Prasad A.S. (2008). Clinical, immunological, anti-inflammatory and antioxidant roles of zinc. Exp. Gerontol..

[508] Fortes C., Forastiere F., Agabiti N., Fano V., Pacifici R., Virgili F., Piras G., Guidi L., Bartoloni C., Tricerri A. (1998). The Effect of Zinc and Vitamin A Supplementation on Immune Response in an Older Population. J. Am. Geriatr. Soc..

[509] Lee S.R. (2018). Critical role of zinc as either an antioxidant or a prooxidant in cellular systems. Oxid. Med. Cell Longev..

[510] Pecora F., Persico F., Argentiero A., Neglia C., Esposito S. (2020). The Role of Micronutrients in Support of the Immune Response against Viral Infections. Nutrients.

[511] Prasad A.S., Bao B., Beck F.W., Kucuk O., Sarkar F.H. (2004). Antioxidant effect of zinc in humans. Free. Radic. Biol. Med..

[512] Xia S., Shen Y., Yu W. (2009). Study on mechanism and intervention of inflamm-aging in rats. Chin. J. Gerontol..

[513] Xia S., Shen Y., Yu W. (2008). Inflamm-aging related genes expression in aged rat hippocampus and Icariin intervention outcome. Geriatr. Health Care.

[514] Zheng J., Hu S., Wang J., Zhang X., Yuan D., Zhang C., Liu C., Wang T., Zhou Z. (2021). Icariin improves brain function decline in aging rats by enhancing neuronal autophagy through the AMPK/mTOR/ULK1 pathway. Pharm. Biol..

[515] Li W.-X., Deng Y.-Y., Li F., Liu B., Liu H.-Y., Shi J.-S., Gong Q.-H. (2015). Icariin, a major constituent of flavonoids from Epimedium brevicornum, protects against cognitive deficits induced by chronic brain hypoperfusion via its anti-amyloidogenic effect in rats. Pharmacol. Biochem. Behav..

[516] Yan N., Wen D.S., Zhao Y.R., Xu S.J. (2018). Epimedium sagittatum inhibits TLR4/MD-2 mediated NF-κB signaling pathway with anti-inflammatory activity. BMC Complement. Altern. Med..

[517] Han G., Zhang C., Qian C., Na M., Ding Y., Zhao H. (2018). Total Flavonoids of Epimedium Reduce Inflammatory Reaction via AMPK/SIRT1/NFκB Signaling Pathway in Testes of Natural Aging Rats. Nat. Prod. Res. Dev..

